# Mechanically robust non-swelling cold water fish gelatin hydrogels for 3D bioprinting

**DOI:** 10.1016/j.mtbio.2025.101701

**Published:** 2025-03-22

**Authors:** Tobias Hammer, Ke Yang, Tobias Spirig, Barbara Meier-Schiesser, Markus Rottmar, Katharina Maniura-Weber, René M. Rossi, Kongchang Wei

**Affiliations:** a Empa, Swiss Federal Laboratories for Materials Science and Technology, Laboratory for Biomimetic Membranes and Textiles, Lerchenfeldstrasse 5, St. Gallen, 9014, Switzerland; b Empa, Swiss Federal Laboratories for Materials Science and Technology, Laboratory for Biointerfaces, Lerchenfeldstrasse 5, St. Gallen, 9014, Switzerland; c University Hospital Zurich, Department of Dermatology, Zürich, Zürich, Switzerland

## Abstract

Three-dimensional (3D) bioprinting of hydrogels allows embedded cells to be patterned and hosted in an extracellular matrix (ECM)-mimicking environment. This method shows great promise for the engineering of complex tissues on account of the facile spatial control over materials and cells within the printed constructs. Hydrogels, which represent extensively explored and employed biomaterials for 3D bioprinting, are characterized by both their high water content and swelling behavior. Post-printing swelling inevitably alters the geometrical and mechanical properties of printed features, thus causing a deviation from the original design and affecting both cellular function and tissue structure. Despite substantial effort being dedicated to the development of non-swelling hydrogels, their application in 3D encapsulation and bioprinting of living cells is yet to be realized, owing to limitations imposed by their often tedious material syntheses and complex network structures. Herein, we describe a new type of non-swelling hydrogel based fully on cold water fish gelatin (cfGel-Hydrogel) consisting of only a single network formed via thiol-ene "click" chemistry. We show that such cfGel-Hydrogels enable 3D patterning of living cells in a shape-retaining and mechanically robust matrix. These cfGel-Hydrogels show negligible swelling (<2 %) under physiologically relevant conditions (simulated by 37 °C PBS buffer), while also being able to withstand large cyclic deformations (80 % compressive strain) by dissipating around 40 % of the imposed loading energy. Human dermal fibroblast (HDF)-laden cfGel-Hydrogels could be fabricated via extrusion-based 3D printing, allowing for the *in vitro* culturing of cells in shape-retaining constructs, thus offering new opportunities for hydrogel-based applications in tissue engineering and regenerative medicine.

## Graphical abstract

**Figure:**
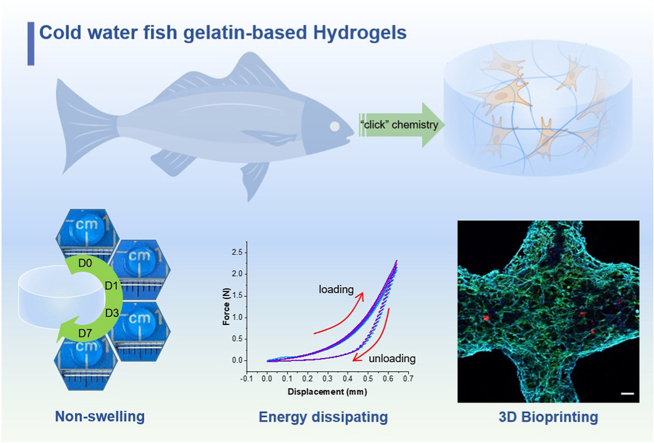

## 1 Introduction

For most contemporary tissue engineering ambitions, the simple combination of cells with biomaterial components is insufficient to yield constructs that can accurately mimic the complex biological structures and functions of the tissues they are designed to recreate [1]. Consequently, three-dimensional (3D) bioprinting has been established as a promising strategy for developing functional living tissue constructs through the precise spatial deposition of biomaterials and living cells [2]. Hydrogels, owing to their hydrated nature and tuneable properties, can be engineered to mimic many aspects of the extracellular matrix (ECM) of native tissues, rendering them excellent candidates for a broad spectrum of biomedical applications [[3], [4], [5]], including 3D printing of living cells for tissue engineering and regenerative medicine [6]. They can support the rapid patterning of cells in pre-defined tissue-mimicking structures prior to *in vitro* cultivation or *in vivo* transplantation [7,8].

However, despite tremendous advances in 3D-printing of cell-laden hydrogel structures, retaining the geometrical and mechanical properties of the original design still remains challenging. While many existing 3D-printable hydrogels are well tuned to support cell encapsulation and cultivation, post-printing volumetric changes (swelling or de-swelling) under physiological conditions could alter their geometrical features and mechanical properties, which could potentially result in undesired structural deficiencies and cellular responses [[9], [10], [11]]. Retaining the designed geometry and original mechanics of 3D-printed cell-laden hydrogel constructs under cell culture conditions over a prolonged period of time represents an important design aspect, which can not be easily achieved without employing complex formulations or crosslinking strategies. For instance, a recent study reported a 3D printed and shape-retaining hydrogel graft for cell culture [12]. The procedure involved a dual bioink 3D printing strategy, where covalently cross-linked poly(ethylene) glycol and ionically cross-linked alginate were required to form a double-network support prior to printing cells with a gelatin methacrylate (GelMA) bioink.

Conventional hydrogels in their as-prepared relaxed state are commonly subject to swelling, featuring conformational extensions of polymer chains and the resultant volumetric expansion of the hydrophilic polymer network [13]. Although swelling can facilitate beneficial effects such as the absorption of physiological fluids (e.g. blood and wound exudate) and transportation of nutrients and metabolites [[14], [15], [16], [17]], it often leads to compromised mechanical properties [[18], [19], [20]], unpredictable macromolecular release dynamics [21,22], cell-matrix interactions and mechanical signaling [23,24], in addition to the previously mentioned structural alterations. When used *in vivo*, swelling may cause undesired compression and potential irritation to surrounding tissues [[25], [26], [27], [28]]. Consequently, various strategies have been proposed to engineer hydrogels with low-, non-, and de-swelling properties over the past decade [13]. For instance, a combination of two synthetic tetra-arm polymers has been used to achieve hydrogels with non-swelling behaviors and preserved mechanical properties under physiological conditions [18]. This was realized by the delicate balance between the extension of the hydrophilic polymer chains and the shrinkage of the thermoresponsive polymer chains of the hydrogel network. Alternatively, the formation of non-osmotic hydrogels has also been achieved by using synthetic tetra-arm copolymer pairs with controlled hydrophobicity [29,30]. While these approaches relied on complex structural designs of synthetic polymers or polymeric networks, other strategies utilizing network reinforcements through interpenetrating double-networks [[31], [32], [33]], multi-crosslinking approaches [34], supramolecular interactions [[35], [36], [37]] and nanocomposites [38,39] were also reported to achieve non-swelling hydrogels.

The non-swelling property of the abovementioned hydrogels originates and heavily relies on the structural tailorability of synthetic polymers and their networks. In contrast, natural biopolymers (e.g. proteins and polysaccharides) possess a limited potential for structural alteration. Consequently, despite their superior biocompatibility and biodegradability as well as their bioactive interactions with living cells, natural biopolymers constitute a substantially less feasible choice in the creation of non-swelling hydrogels compared to their synthetic counterparts. In the past years, Li H. et al., have intensively explored the potential of fully protein-based hydrogels in mimicking mechanical aspects of natural muscle tissue [40]. By combining folded domains and unstructured sequences engineered into titin-mimicking artificial elastomeric proteins, they succeeded in generating soft hydrogel biomaterials that demonstrated muscle-like behavior with high resilience and effective energy dissipation. Introduction of chain entanglements allowed these engineered protein-based hydrogels to become highly stiff and tough following de-swelling under physiological conditions [41]. These studies demonstrate the potential of utilizing conformational folding of designed proteins in conveying both a reinforced structure and non-swelling properties onto purely protein-based hydrogels. Further development could potentially lead to applications in 3D bioprinting of cells within engineered protein matrices.

Different from engineered proteins, whose production often requires recombinant technologies, gelatin constitutes a hydrolytic product of collagen. Being derived from the primary structural protein of the mammalian ECM, gelatin retains many of collagen's important characteristics, including the arginine-glycine-aspartate (RGD) sequence, an integrin-binding domain that is instrumental for cell adhesion and for conferring biological functionality to the hydrogel matrix [[42], [43], [44]]. This cell-adhesive feature distinguishes gelatin from many other natural polymers (e.g., hyaluronic acid, alginate, and others) that require chemical modification to provide cell-adhesive functions. Therefore, as a naturally abundant, cell-interactive and cost-efficient biopolymer, gelatin is particularly attractive for the development of mechanically robust and non-swelling hydrogels that may advance the field of bioengineering and regenerative medicine [40,41,45,46].

While gelatin from mammalian sources represents the most commonly used variant, cold water fish gelatin (cfGel) has recently emerged as a promising alternative owing to its lower gelling and melting temperatures, which allow for dissolution and processing at ambient temperatures, as well as a low immunogenicity and risk of disease transmission in combination with fewer sociocultural concerns regarding its use [47,48]. Furthermore, due to molecular features such as the lower content of proline and hydroxyproline in cfGel compared to mammalian gelatin [47], the molecular mobility of polypeptides between hydrogel crosslinking points of cfGel has been found to be higher than that of the latter [49], representing an important factor for improving cell-matrix remodeling rates and facilitating stem cell migration in gelatin hydrogels [49]. Owing to these properties, cold water fish gelatin has already seen applications in the engineering of various tissues such as bone [50], cartilage [51,52], and skin [53].

**Fig. 1:**
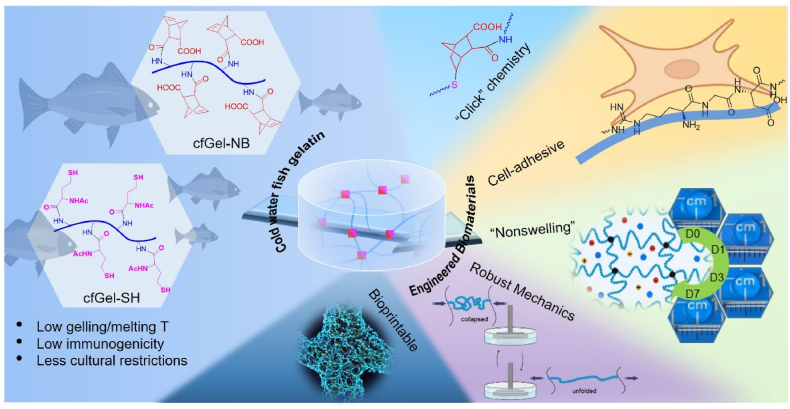
Design and features of a fully cold water fish gelatin-based hydrogel (cfGel-Hydrogel).

Some of the reported strategies to facilitate the formation of hydrogels from cfGel involve enzymatic crosslinking in the presence of transglutaminase (TGase) [54], photoinitiated chain polymerization of cold water fish-derived GelMA [49,55,56] or thiol-ene "click" crosslinking between norbornene-functionalized cfGel (cfGel-NB) and tetra-thiol polyethylene glycol (4-arm PEG-SH) [56]. However, many of these strategies suffer from drawbacks pertaining to the lack of control over the spatiotemporal gelation of the hydrogels (enzymatic crosslinking), heterogeneous network structures (GelMA) or the involvement of synthetic components and two-component matrices (e.g. 4-arm PEG-SH) that could compromise their potential for biomedical applications [57]. Herein, we describe a fully cold water fish gelatin-based hydrogel (cfGel-Hydrogel) composed of only a single network formed via thiol-ene "click" chemistry (Fig. 1). Under physiological conditions, such cfGel-Hydrogels showed non-swelling behavior and robust mechanical properties. They were capable of withstanding large cyclic deformations while simultaneously featuring rapid mechanical recovery and notable energy-dissipation. In combination with excellent cytocompatibility, these properties make cfGel-Hydrogels a promising platform for the development of shape retaining cell-laden constructs, which was demonstrated by using extrusion-based 3D bioprinting in this study and could contribute to the advancement of tissue engineering and regenerative medicine in the future.

## 2 Results and discussion

### 2.1 Synthesis and rheological characterization of cfGel-Hydrogels

**Fig. 2:**
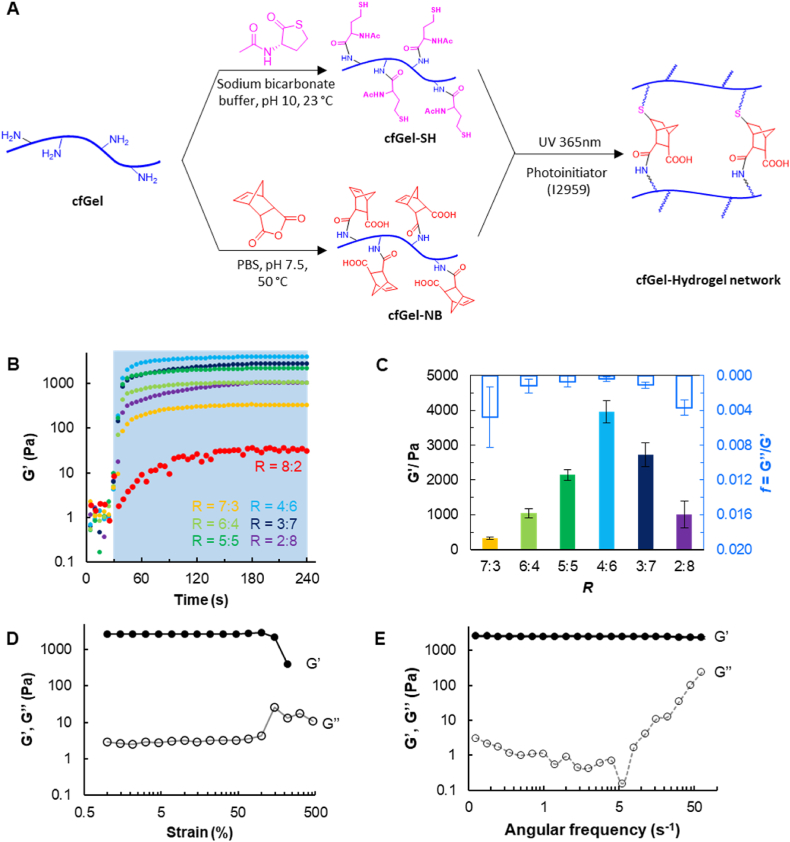
(**A**) Synthesis and "click" crosslinking of functional cold water fish gelatin polymers (cfGel-SH and cfGel-NB). (**B**) Representative rheological time-sweep measurements of cfGel-Hydrogels with the same content of polymer (5 wt%) but varied weight ratios between cfGel-NB and cfGel-SH polymers (R). Blue area indicates period of UV-exposure. (**C**) Rheological properties of cfGel-Hydrogels with different R, *n = 3*. (**D**) Representative rheological strain-sweep measurement of 5 wt% cfGel-Hydrogels (R = 5:5). (**E**) Representative rheological frequency-sweep measurement of 5 wt% cfGel-Hydrogels (R = 5:5). (For interpretation of the references to colour in this figure legend, the reader is referred to the Web version of this article.)

To develop a fully cfGel-based hydrogel, we synthesized two functional polymers (Fig. 2A, see also Methods in Supporting Information), namely cfGel-10.13039/100004395NB and thiol-functionalized cfGel (cfGel-10.13039/100024172SH), both of which inherit the intrinsically bioactive characteristics of cfGel while also conveying rapid "click" crosslinking. The content of functional groups was found to be ∼170 μmol norbornene [-NB] groups per gram polymer for cfGel-NB (^1^H NMR, Fig. S1), and ∼241 μmol thiol [-SH] groups per gram polymer for cfGel-SH (Ellman's reagent assay, Fig. S2). It is worth mentioning that synthesizing two different gelatin polymers with a singular modification each enables a better control over the final polymeric composition and network structure of the resultant hydrogels compared to the widely used GelMA formulations [58]. This approach also allowed us to avoid commonly used DTT or PEG-based crosslinkers, resulting in "click" hydrogels with biochemically homogeneous network structures [59]. Upon UV exposure (*λ* = 365 nm, 10 mW/cm^2^), cfGel-Hydrogels formed rapidly within ∼10 s (Fig. 2B), demonstrating the excellent crosslinking efficiency of thiol-ene "click" reactions between cfGel-NB and cfGel-SH. The rapid curing of cfGel-Hydrogels was not affected by the mass ratio between cfGel-NB and cfGel-SH, as revealed by oscillatory time-sweep rheological measurements (Fig. 2B and Fig. S3). The ratios between cfGel-NB and cfGel-SH (R, by weight) tested include R = 8:2, 7:3, 6:4, 5:5, 4:6, 3:7 and 2:8. For all tested formulations (constant total polymer concentration of 5 wt%) with different R, a sharp increase in G′ within the first ∼10 s following UV-light exposure was observed, except for R = 8:2, which lacked a sufficient amount of thiol groups to facilitate crosslinking of cfGel polymers into a 3D network and was thus excluded from further analyses (Fig. 2B). This R-independent rapid gelation (Fig. S3) represents one of the characteristics of "click" crosslinking and the resulting step-growth polymerization.

By adjusting R, the stiffness of cfGel-Hydrogels could be altered without changing the overall composition (5 wt% cfGel polymer) or affecting the rapid curing feature. A concomitant increase of G' (storage modulus) was observed as the amount of cfGel-SH increased (R from 7:3 to 4:6). Beyond this range (R from 3:7 to 2:8), excessive cfGel-SH led to a reduction in G' (Fig. 2C). This marks a dissimilarity to GelMA-based hydrogels, which usually require different degrees of chemical functionalization to achieve a difference in stiffness if the polymer concentration is fixed. It is noteworthy that the best R for the highest G′ was found to be 4:6. However, based on our quantification of the degree of functionalization for each polymer, the optimal ratio (R) was expected to be around 6:4, which ensures the correct [-10.13039/100004395NB]:[-10.13039/100024172SH] stoichiometry (Supporting information Section 2.4). This deviation suggests the formation of additional disulfide bonds between excessive thiol moieties (Fig. S4), which could result in additional crosslinking between cfGel polymers [60].

As the "click" crosslinking provides a homogeneous network structure, the excellent structural strength of such cfGel-Hydrogels was demonstrated by the broad linear viscoelastic (LVE) range (Fig. 2D). The formed hydrogels were capable to withstand strains up to 100 % without incurring structural damage or breaking, as revealed by oscillatory strain-sweep measurements. It was observed that the drop in modulus at high strain levels was due to adhesive failure between the parallel plate geometry of the rheometer and the hydrogels, rather than due to cohesive failure of the gels themselves. This non-brittle characteristic was demonstrated by all formulations with total cfGel polymer concentrations ranging from 5 wt% to 10 wt% (Fig. S5). Furthermore, their viscoelastic behavior under small oscillatory shear strain (*r* = 1 %) featured very low damping, with damping factors *f* ≪ 1 (Fig. 2C), suggesting that the homogeneous single-network matrix exhibits good resistance to plastic deformation. Frequency-sweep data further revealed such low-damping behavior until the angular frequency reached 5 s^−1^, (Fig. 2E, and Fig. S5-B). At higher frequencies, an increase in G″ was observed, indicating insufficient relaxation of the cfGel network under fast shearing.

### 2.2 Non-swelling property of cfGel-Hydrogels under physiological conditions

**Fig. 3:**
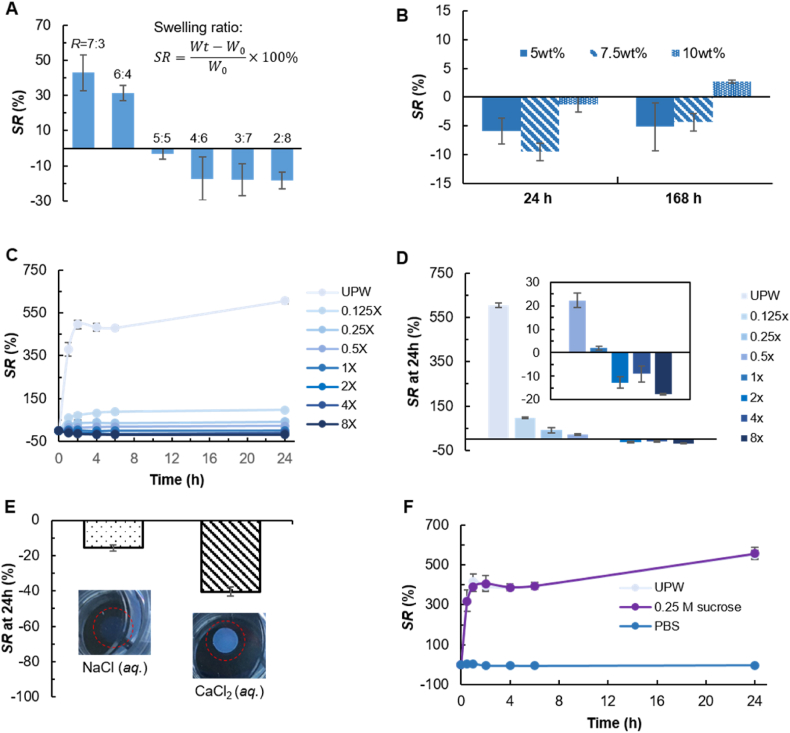
**Swelling behavior of cfGel-Hydrogels.** (**A**) Swelling ratio (SR) of cfGel-Hydrogels consisting of various polymer weight ratios between cfGel-NB and cfGel-SH (R) after 24 h, *n = 3*. (**B**) Swelling ratio of cfGel-Hydrogels (R = 5:5) with different total polymer concentrations after incubation in 37 °C PBS for 24 h and 168 h, *n = 3*. (**C**) Degree of swelling of cfGel-Hydrogels in the presence of PBS at various concentrations compared to ultrapure water (UPW). (**D**) Comparison of the swelling ratio of each condition after 24 h. Inset shows the range from 8 × to 0.5 × , *n = 3*. (**E**) Swelling behavior of cfGel-Hydrogels in solutions of NaCl and CaCl_2_, *n = 3*. (**F**) Swelling of cfGel-Hydrogels in isotonic solutions of sucrose (0.25 M) in comparison to UPW and PBS, *n = 3*.

As shown in Fig. 3A, cfGel-Hydrogels composed of a fixed initial cfGel polymer content (5 wt%) with R ranging from 7:3 to 2:8 exhibited limited swelling or de-swelling, characterized by a swelling ratio (SR) within 50 % under physiologically relevant conditions (in PBS, 37 °C). We found that formulations with R = 7:3 and R = 6:4 experienced the highest swelling ratio (SR: 42.9 ± 10.1 % and 31.5 ± 4.3 %, respectively). While shifting towards cfGel-SH-dominant ratios (R = 4:6, 3:7 and 2:8), hydrogels experienced a concomitant decrease in weight due to de-swelling (SR: −17.3 ± 12.4 %, −17.9 ± 8.9 % and −18.2 ± 4.8 %, respectively). Notably, hydrogels with R = 5:5 exhibited only a negligible weight change after 24 h (SR: −3.2 ± 3.1 %), making them essentially non-swelling. Along with the decrease of R from 7:3 to 4:6, an accompanying decrease of swelling was observed, which is in accordance with the increase in G′ of the as-prepared hydrogels (Fig. 2C, and Fig. S6-A), which implicated increased crosslinking densities. Interestingly, while a further decrease in R caused a reduction in G′ due to the insufficient amount of cfGel-NB to mediate effective crosslinking, it did unexpectedly not result in increased swelling. Formulations with comparable G' (R = 6:4 and R = 2:8) displayed different swelling behaviors, implying that although the overall makeup of the hydrogels remained the same (5 wt% cfGel with similar crosslinking density), the difference in residual functional groups ([-NB] or [-SH] groups) in as-prepared hydrogels can influence their swelling behaviors. This suggested that although residual [-SH] groups did not contribute to rapid thiol-ene crosslinking (initial G′ of the as-cured cfGel-Hydrogels), they could induce post-curing slow formation of additional crosslinks (e.g. disulfide bonds), thereafter affecting the swelling behavior. It should be further noted that albeit the swelling ratios of 4:6, 3:7 and 2:8 demonstrate similar values, their respective SD are comparatively high.

Regardless of variations, hydrogels prepared from all tested formulations showed a limited capacity for swelling (Fig. 3A). We chose to perform subsequent studies with the formulation R = 5:5, as it demonstrated only a negligible change in weight under physiologically relevant conditions, thus featuring the non-swelling properties desired for retaining hydrogel geometry and mechanics. Therefore, unless stated otherwise, the term "cfGel-Hydrogel" will henceforth refer to hydrogels with R = 5:5. As shown in Fig. 3B, the non-swelling behavior was not affected by the total cfGel polymer concentration. cfGel-Hydrogels prepared with 5 wt%, 7.5 wt% and 10 wt% polymer precursors showed similar swelling behavior (SR < ±10 %) after 168 h of incubation in PBS (Fig. S6-B). To gain more insight into the underlying mechanism that drives the swelling behavior of cfGel-Hydrogels, we performed a series of swelling studies in PBS solutions of varying concentrations, ranging from 8 × (with 8 times higher salt concentration than purchased PBS) to 0.125 × , as well as ultrapure water (UPW) (Fig. 3C). After 24 h, cfGel-Hydrogels exposed to concentrated solutions of PBS exhibited negative swelling (SR = −17.7 ± 0.4 %, −9.0 ± 3.4 % and −12.7 ± 2.4 % for 8 × , 4 × and 2 × PBS respectively, Fig. 3D). In contrast, cfGel-Hydrogels showed increased swelling in PBS solutions of lower ionic strength (≤1 × ), with SR = 1.9 ± 0.7 %, 22.2 ± 3.0 %, 41.7 ± 12.1 %, and 96.4 ± 3.6 % for 1 × , 0.5 × , 0.25 × , and 0.125 × respectively.

In sharp contrast to their swelling behavior in PBS, cfGel-Hydrogels experienced significant degrees of swelling in UPW, reaching a SR of 605.5 ± 11.0 %, around 5-fold higher than what was observed in 0.125 × PBS. This indicated that the presence of even a small amount of salts can significantly restrict the swelling of cfGel-Hydrogels. We further found this phenomenon to occur irrespective of pH values. Without adjusting the pH of UPW (5.83), the addition of NaCl (0.14 M) or CaCl_2_ (0.07 M) demonstrated comparative effectiveness to PBS (pH 7.5) in reducing the swelling of cfGel-Hydrogels (Fig. 3E). While we observed similar swelling ratios of cfGel-Hydrogels in NaCl (SR = −15.7 ± 1.8 %) compared to that in PBS, cfGel-Hydrogels exposed to CaCl_2_ demonstrated a more pronounced de-swelling (SR = −40.4 ± 2.6 %), accompanied by a greater reduction in volume and the adoption of an opaque appearance. This could be due to the interaction between divalent Ca^2+^ ions and the carboxylic groups of gelatin, which resulted in a reduced number of free carboxylic acid groups available for hydrogen bonding with the surrounding water molecules [61]. These additional interactions can further facilitate polymer chain aggregation within the hydrogels [61], which leads to the observed decrease in transparency.

It was recently reported that ionic strength could have a significant impact on conformational fluctuations of gelatin polymers in solutions [62]. Herein, we demonstrate that the responsiveness to salts is also present in cfGel polymers crosslinked into a 3D network, which could be explained by the osmotic pressure-mediated flow between the latter's interior and exterior environment [63,64]. We hypothesize that PBS containing higher salt concentrations (>1 ×) elicited negative swelling by extracting water from the interior of the hydrogel, whereas PBS with lower salt concentrations (<1 ×) had the opposite effect. To validate this hypothesis, we exposed cfGel-Hydrogels to 1 × PBS, UPW and isotonic (0.25 M) solutions of sucrose (Fig. 3F). Both PBS and 0.25 M sucrose constitute isotonic media, thus neither of them should elicit any osmotic pressure based solely on the difference in solute-concentration between the exterior and interior of the gels. The distinguishing factor in this case is the low ionic strength of the sucrose solution as opposed to PBS. Our data shows that swelling of cfGel-Hydrogels in an isotonic sucrose solution is comparable to that in UPW. This indicates that altering the chemical potential of a solution by increasing the concentration of solutes does not affect the swelling behavior of cfGel-Hydrogels as long as there is no concomitant increase in ionic strength. Instead, the swelling behavior of cfGel-Hydrogels appears to be closely tied to polymeric conformational responses of the network to the ionic content of its surrounding medium.

Both gelatin in general and fish gelatin specifically have been reported to experience salt concentration-dependent structural, physicochemical and mechanical alterations when exposed to monovalent or polyvalent salts [65]. Similarly, conventional porcine gelatin-derived GelMA hydrogels were found to swell significantly less in PBS than in water (Fig. S7-A) However, after 48 h of incubation in PBS (Fig. S7-B), the mass change of GelMA-based hydrogels (SR = −32.1 ± 13.3 %) was more profound than for cfGel-Hydrogels (SR = −6.1 ± 3.2 %). Conformational fluctuations are attributed to the changing dynamics between the electrostatic interactions of salt ions and the charged polymer backbone, altering the screening-off of long- and short-range electrostatic repulsions and effectuating tighter or looser association of gelatin α-chains [47,62,66]. Furthermore, as the concentration of salt increases, competition for water molecules among the ionic and polymeric constituents causes a series of transitions in the hydration state of gelatin, accompanied by both chain shrinkage and expansions [67]. When salt concentrations reach a certain threshold, electrostatic interactions can become strong enough to break and restructure hydrogen bonds and alter the secondary structure of gelatin [68]. Apart from the concentration, the type of salt ion and its location in the Hofmeister series can marginally determine the solubility of gelatin and other proteins in aqueous solutions [69,70]. The introduction of physical crosslinks through the salting-out effect mediated by kosmotropic ions such as ammonium sulfate has been previously employed to increase the mechanical properties of pre-formed gelatin networks by removing the hydration waters of polymer chains and causing their folding or precipitation [71,72]. It is noteworthy that we do not observe SR fluctuations of cfGel-Hydrogels with increasing salt concentrations even though changing electrostatic dynamics reportedly facilitate transitions between polymer chain shrinkage and expansion. Furthermore, we observed the most significant degree of de-swelling accompanied by a loss of transparency for cfGel-Hydrogels incubated in CaCl_2_, despite the chaotropic nature of Ca^2+^ presumably promoting the chain expansion effect. This implies that gelatin conformational responses to salt concentrations behave differently in a network structure compared to those in a solution state.

### 2.3 Robust mechanical properties of cfGel-Hydrogels

**Fig. 4:**
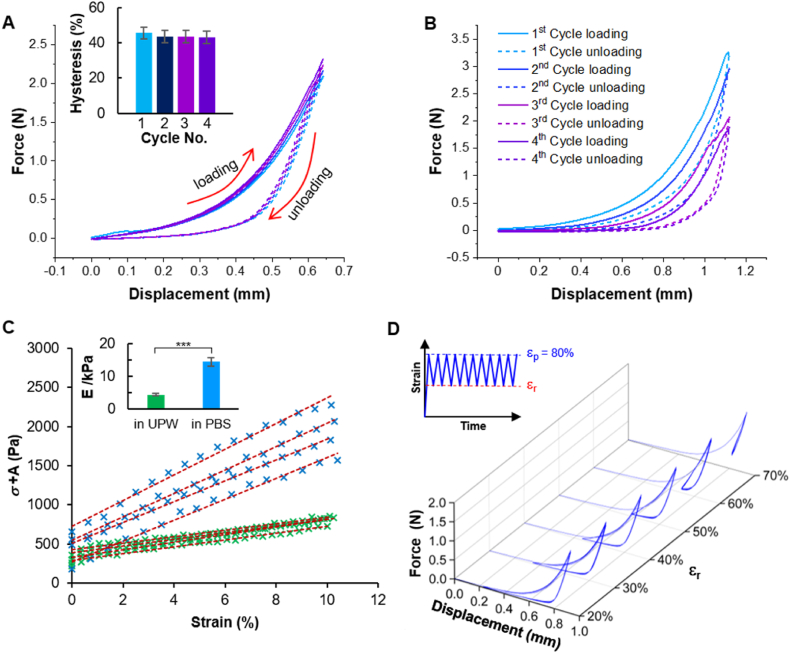
Cyclic compressive test of cfGel-Hydrogels in PBS (37 °C) (**A**) and UPW (37 °C) (**B**). Hysteresis is presented as mean ± s.d. *n = 3*; panel A and B share the same legends. (**C**) Stress-strain curves for determining Young's modulus (inset) of cfGel-Hydrogels with linear fitting (stress values were adjusted for visualization purposes; A_PBS_ = 1000, A_UPW_ = 0). ∗∗∗P < 0.001 (**D**) Cyclic compressions of cfGel-Hydrogels in PBS (37 °C) under various residual strains (ε_r_).

Based on previously established knowledge on the benefits of restricted swelling for the preservation of hydrogel mechanical strength [18], we expected cfGel-Hydrogels subjected to physiologically relevant conditions to demonstrate superior mechanics by virtue of their non-swelling behavior compared to their swollen counterparts exposed to UPW. As revealed by cyclic compressive tests, cfGel-Hydrogels were able to withstand multiple cycles of high-strain level compressions (80 %) in PBS, without incurring cohesive failure or visible signs of damage (Fig. S8). The mechanical robustness under high-strain compressions was further confirmed by the overlap of consecutive loading curves (Fig. 4A). Importantly, compressive cycles were performed in close succession without any intermediate waiting times. The observation of nearly identical loading curves indicates an instantaneous recovery of the hydrogel network structure upon unloading. Moreover, cfGel-Hydrogels in PBS consistently exhibited high levels of hysteresis (>40 %) between consecutive loading-unloading cycles, thus revealing their capacity for dissipating massive loading energies and preventing structural failure (Fig. 4A, inset). In contrast, cfGel-Hydrogels that experienced swelling as a consequence of prior exposure to UPW were not capable of withstanding the same levels of compressive deformation but instead suffered cohesive failure and structural breakdown (Fig. 4B). Similar phenomena were also found in conventional GelMA-based hydrogels in PBS (Fig. S9-A). However, after swelling in UPW, GelMA-based hydrogels exhibited less tolerance to compressive forces due to an increase in brittleness, as indicated by the significant difference between the first and second loading curves (Fig. S9-B). Our results are in line with previous reports on the detrimental effects of swelling on the mechanical properties of hydrogels and further emphasize on the importance of the non-swelling behavior exhibited by cfGel-Hydrogels in tolerating excessive deformation [18].

The restricted swelling of cfGel-Hydrogels in PBS suggests the existence of unexpanded cfGel polymer chains providing the hydrogel network with a higher apparent crosslinking density (N ∝ 1/M_c_) compared to cfGel-Hydrogels in UPW, where N is the crosslinking density (numbers of elastically effective polymer strands per unit volume), and M_c_ is the apparent average molecular weight (Mw) of elastically effective polymer strands in between two neighboring crosslinking points. According to the rubber elasticity theory, the Young's modulus (E) of a chemically crosslinked cfGel-Hydrogel should be inversely proportional to M_c_ (E ∝ 1/M_c_). Experimental measurements confirmed the higher Young's moduli of cfGel-Hydrogels in PBS (E_PBS_) compared to UPW (E_UPW_), with E_PBS_ (14.5 ± 1.3 kPa) being around three times higher than E_UPW_ (4.4 ± 0.3 kPa) (Fig. 4C).

Depending on their intended application, hydrogels might not experience a complete relaxation during consecutive instances of mechanical stress. Taking this factor into consideration, it is important for such hydrogels to be able to resist mechanical deterioration during cyclic deformations where residual strains might remain. Subjecting cfGel-Hydrogels in PBS to cyclic compressions with various residual strains (Fig. 4D, insert) yielded identical peak forces for subsequent cycles as observed for the first loading curve (at peak strain ε_p_ = 80 %). Additionally, consecutive loading-unloading curves were found unaltered under all residual strains (ε_r_ = 20–70 %) (Fig. 4D). Conversely, the degree of hysteresis was dependent on the strain imposed on the hydrogels during cyclic deformation (Δε = ε_p_ - ε_r_), showing more pronounced hysteresis with a concomitant increase of Δε. For ε_r_ = 70 % (Δε = 10 %), only minor hysteresis was observed. Lowering residual strains down to 60 % (Δε = 20 %) already resulted in a considerably higher hysteresis, indicating substantial energy dissipation and recovery. The ability of cfGel-Hydrogels to withstand extended periods of non-resting compressive stress without suffering debilitating mechanical performances constitutes an essential attribute for their potential use in biomedical applications involving excessive dynamic loadings [73]. In addition, cfGel-Hydrogels can also withstand uniaxial stretching cycles, showing no signs of fatigue during repeated cycles of stretching with tensile strains up to 168 % (Fig. S10).

**Fig. 5:**
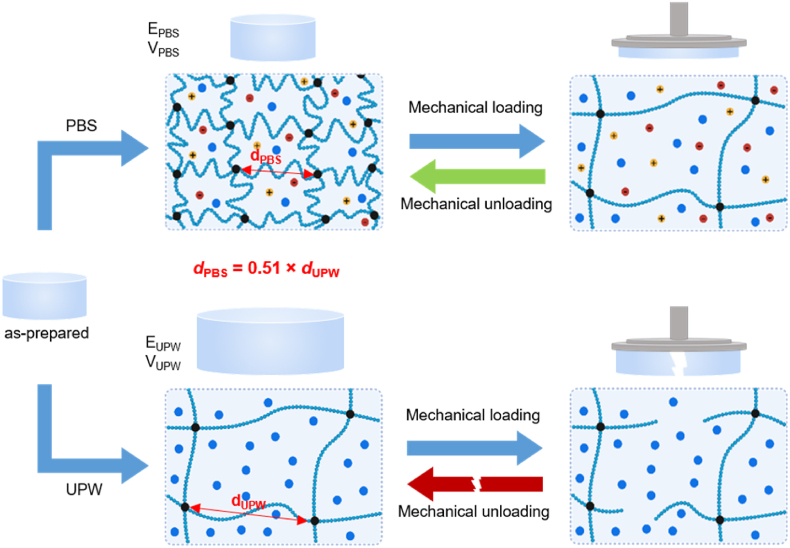
Schematic illustration of the proposed molecular mechanism supporting the observed swelling and mechanical properties exhibited by cfGel-Hydrogels in either PBS or UPW.

The effect of restricted swelling on the mechanical properties of cfGel-Hydrogels can be explained by the conformational change of cfGel polymer chains. Upon compressive loading in PBS, forced chain expansions dissipate the loading energy and prevent the rupture of the polymer network (Figs. S8–C). The conformational retraction of the polymer chains upon unloading rapidly restores the original state of the 3D cfGel polymer network structure, thus giving rise to both the energy-dissipating and fast-recovering properties we observed for cfGel-Hydrogels in PBS (Fig. 5). To reveal the conformational shrinkage of cfGel polymers in PBS compared to those in UPW, we defined the averaged distance between two neighboring crosslinking points as *d*. According to the rubber elasticity theory, the relative change of cfGel-Hydrogel Young's modulus in different swelling media can be expressed as:

$$\frac{E_{\text{UPW}}}{E_{\text{PBS}}}=\frac{M_{C-\text{PBS}}}{M_{C-\text{UPW}}}$$

where M_c-PBS_ and M_c-UPW_ are the apparent average M_w_ of polymer strands in between two crosslinking points in PBS and UPW respectively. As *d* can be correlated to M_c_ as (*d* ∝ M_c_^0.57^) [74], it can be estimated that:

$$\frac{d_{\text{PBS}}}{d_{\text{UPW}}}={\left(\frac{M_{C-\text{PBS}}}{M_{C-\text{UPW}}}\right)}^{0.57}={\left(\frac{E_{\text{UPW}}}{E_{\text{PBS}}}\right)}^{0.57}={\left(\frac{4.4\;\text{kPa}}{14.5\;\text{kPa}}\right)}^{0.57}=0.51$$

where *d*_UPW_ and *d*_PBS_ are the distances between two neighboring crosslinking points in UPW and PBS, respectively. This reflected the substantial conformational shrinkage (49 %) of cfGel polymer chains in PBS compared to those in UPW (Fig. 5), which is key to the abovementioned energy dissipating and rapid recovery properties of cfGel-Hydrogel in PBS.

Moreover, the equilibrated volume of cfGel-Hydrogels in UPW compared to that in PBS can be estimated as below:

$$\frac{V_{\text{UPW}}}{V_{\text{PBS}}}={\left(\frac{d_{\text{UPW}}}{d_{\text{PBS}}}\right)}^{3}={\left(\frac{1}{0.51}\right)}^{3}=754\%$$

where V_UPW_ and V_PBS_ is the equilibrated volume of cfGel-Hydrogels in UPW and PBS respectively. This is close to our observed swelling behavior (Fig. 3D), where W_PBS_ = 1.02 x W_0_, and W_UPW_ = 7.06 x W_0_, thus giving rise to the observed value of W_UPW_/W_PBS_ = 692 %. Considering the high water content of the hydrogels (initially 95 wt%), their overall density should be close to that of water, therefore V_UPW_/V_PBS_ can be estimated as the measured W_UPW_/W_PBS_.

### 2.4 3D encapsulation and bioprinting of human dermal fibroblasts

**Fig. 6:**
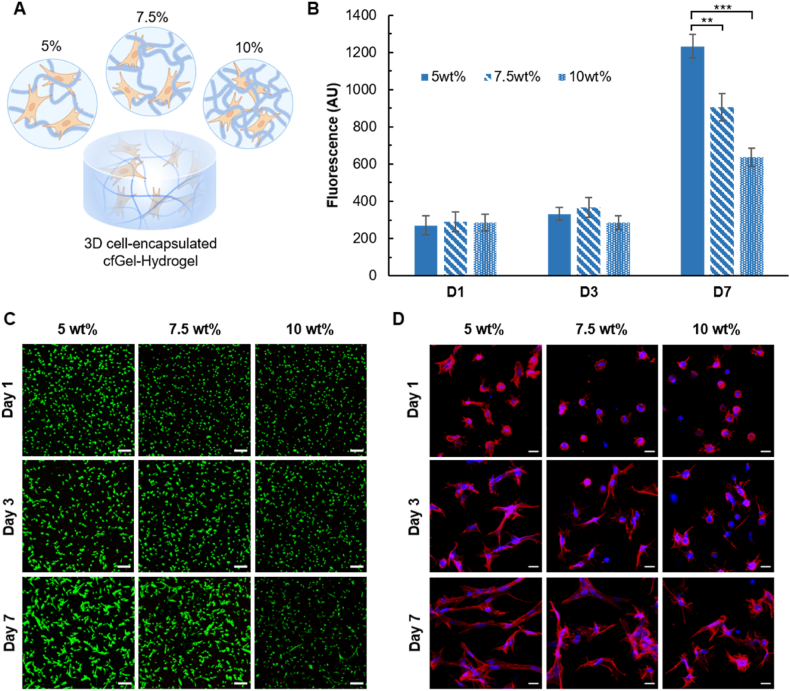
**3D cell encapsulation and cytocompatibility assessment.** (**A**) Schematic representation of the encapsulation of cells in cfGel-Hydrogels with different total polymer concentrations (R = 5:5). (**B**) Metabolic activity of encapsulated HDFs at different time points as determined by Alamar Blue assay (*n = 3*, ∗∗P < 0.01, ∗∗∗P < 0.001). (**C**) Live/dead images of encapsulated HDFs. Scale bars = 200 μm. (**D**) Immunofluorescence images of encapsulated HDFs stained for actin and nuclei. Scale bars = 20 μm. (For interpretation of the references to colour in this figure legend, the reader is referred to the Web version of this article.)

Given the bioactive properties of a fully gelatin-based matrix in tandem with the mild reaction conditions inherent to the thiol-ene click reaction [75], 3D encapsulation and culturing of living cells within cfGel-Hydrogels was realized. This distinguishes cfGel-Hydrogels from existing non-swelling hydrogels made from fully synthetic polymers [18], or those fabricated via multi-step procedures [41]. Enzymatic degradation studies using Collagenase II (∼2 U/ml) revealed similar degradation times for both cfGel-Hydrogels and GelMA-based hydrogels (Fig. S11), which is important for the spreading of encapsulated cells within the matrix. Primary human dermal fibroblasts (HDFs) were encapsulated in cfGel-Hydrogels by UV-curing of cell-suspended precursor solutions with cfGel polymer concentrations of 5 wt%, 7.5 wt% or 10 wt% (Fig. 6A). Metabolic activity of encapsulated HDFs showed only minor differences within the first three days of culture between hydrogels prepared from either cfGel polymer concentration, indicating an initially limited cell proliferation (Fig. 6B). After seven days, HDFs demonstrated significantly elevated levels of metabolic activity in all hydrogels when compared to day three, as well as significantly higher values with decreasing polymer concentrations. These observations could be substantiated by a live/dead assay and immunofluorescence staining for the actin cytoskeleton, which revealed a high cell viability and more pronounced spreading behavior of HDFs in 5 wt% and 7.5 wt% cfGel-Hydrogels compared to 10 wt% (Fig. 6C). We attribute this to the more restrictive environment imposed by the stiffer network structure of 10 wt% gels, which offers increased resistance to cell spreading and proliferation. That aside, the majority of cells were found viable in all examined cfGel-Hydrogels for the entire cultivation period, thus emphasizing the cytocompatible nature of both the material components and the fabrication method.

**Fig. 7:**
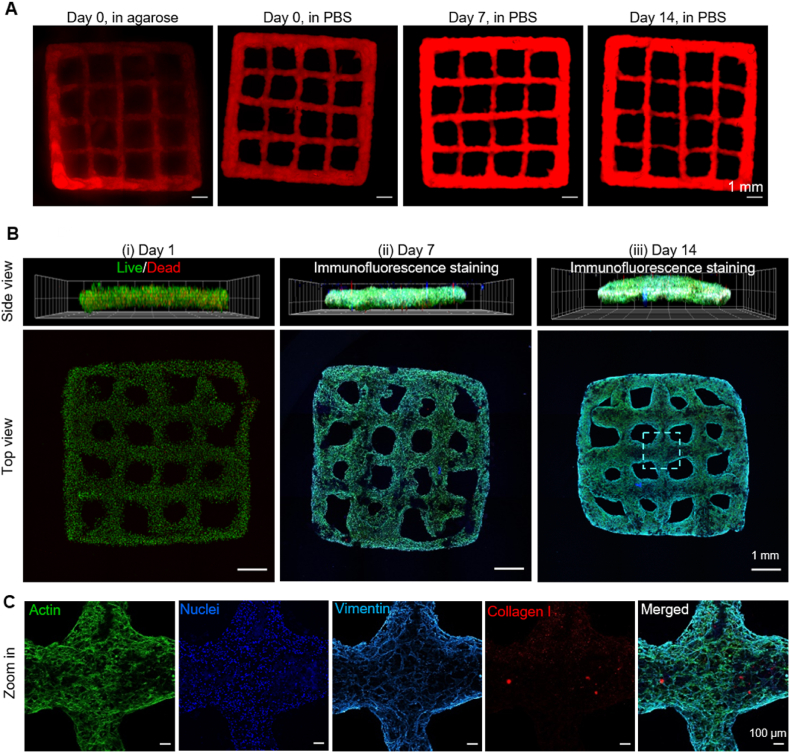
**Cell-free and cell-inclusive 3D printing of cfGel-Hydrogels.** (**A**) Post-printing images of rhodamine B-containing cell-free cfGel-Hydrogels directly after printing (suspended in agarose) and after incubation in DPBS (37 °C) for up to 2 weeks. Scale bars = 1 mm (**B**) Live/Dead and immunofluorescence images of cell-laden printed constructs. (**C**) Magnified section (centre of the "Top view" image in panel B-iii) of printed construct on day 14.

The abovementioned collective properties of cfGel-Hydrogels make them a promising material for 3D bioprinting, especially with regard to cell culturing within shape-retaining 3D constructs. Based on the results of the 3D cell encapsulation, we chose 5 wt% cfGel as the polymer concentration for our printing studies. Due to the low viscosity of the precursor solutions (Fig. S13), we employed freeform reversible embedding of suspended hydrogels (FRESH) for 3D printing, whereby bioinks are directly deposited into viscous support baths [76]. We opted for the use of an agarose support bath formulation in accordance with previous descriptions on account of its non-toxic nature and ease of post-printing removal [77]. For our target structure we chose a simple multi-layered grid (9 mm length × 9 mm width × 0.6 mm thickness), as it would allow us to easily detect dimensional alterations to the individual filaments over time. The printed grids displayed no visually significant structural alterations or noticeable signs of degradation when compared to their as-printed morphology during incubation under physiologically relevant conditions for up to two weeks (Fig. 7A). Measuring and comparing the area of imaged grids using QuPath image analysis software revealed the shape-retaining property of the 3D printed construct (Fig. S12-A) [78].

We subsequently evaluated the effects of the printing of cfGel-Hydrogels on the viability of incorporated HDFs. While equipped with the same features as their cell-free counterparts, dimensions of cell-containing grids were altered to have an increased thickness and a smaller circumference (6 mm length × 6 mm width × 1 mm thickness). A semi-quantitative analysis showed a slightly reduced printing performance compared to cell-free printing (Fig. S14) [79]. Live/dead imaging of cells after both one day and one week post-printing culturing showed no detrimental influence of the printing process on cell viability (Fig. 7B–i, and Fig. S12-B). This aspect likely benefitted from the low viscosity of the cfGel polymer precursor solutions, which reduced shear forces exerted onto cells during extrusion. Moreover, we did not observe major alterations to the shape of the printed structures in the presence of HDFs after one week of culturing (Fig. 7B–ii). However, we did observe some degree of curving or bending of the printed grids by day 14, which might be due to the contractile forces exerted by the incorporated fibroblasts (Fig. 7B–iii). Staining for actin, nuclei and vimentin revealed 3D spreading and homogeneous distribution of collagen I-depositing HDFs throughout the printed structures (Fig. 7C). While the contractile forces exerted by encapsulated cells could be visualized by curving or bending of the cell-laden grids, structural integrity and overall shape of the 3D bioprinted constructs, including more detailed features such as individual filaments and gaps, were maintained even after 14 days of culture (Fig. S12-C). Therefore, as an intrinsically non-swelling material platform, cfGel-Hydrogels offer new opportunities for investigating long-term cell-matrix interactions with minimum interference from swelling.

## 3 Conclusion

To overcome the current challenges faced by mechanically robust and shape-retaining cytocompatible hydrogels in the field of 3D bioprinting, we developed a fully cold water fish gelatin-based "click" hydrogel (cfGel-Hydrogel) composed of a single-network structure that can be formed via simple one-step gelation while also demonstrating significant resistance to swelling under physiologically relevant conditions. The non-swelling characteristic, resulting from salt-induced conformational shrinkage of the gelatin polymer, imparts energy dissipating and rapid recovery properties to cfGel-Hydrogels. The excellent cytocompatibility allows for their application in 3D cell encapsulation and long-term culturing. As a proof-of-concept, their potential application in biofabrication was demonstrated with extrusion-based 3D bioprinting to showcase the resistance of the printed cell-laden structures to media-related swelling and the preservation of the initial scaffold design during post-printing cultivation. It is noteworthy that thiol-norbornene "click" hydrogels have proven themselves as ideal materials for lithography-based high-resolution 3D bioprinting technologies, including digital light projection (DLP) and two-photon polymerization (2 PP) bioprinting [[80], [81], [82]]. Therefore, the "click" crosslinking feature of cfGel-Hydrogels makes them a promising platform for precise engineering of 3D artificial tissues in regenerative medicine and disease modelling [[83], [84], [85], [86]].

## CRediT authorship contribution statement

**Tobias Hammer:** Writing – review & editing, Writing – original draft, Visualization, Validation, Methodology, Investigation, Formal analysis, Data curation, Conceptualization. **Ke Yang:** Writing – review & editing, Validation, Methodology. **Tobias Spirig:** Writing – review & editing, Validation, Investigation, Formal analysis, Data curation. **Barbara Meier-Schiesser:** Writing – review & editing, Validation. **Markus Rottmar:** Writing – review & editing, Visualization, Validation, Methodology. **Katharina Maniura-Weber:** Writing – review & editing, Visualization, Validation, Resources. **René M. Rossi:** Writing – review & editing, Visualization, Validation, Supervision, Resources, Project administration, Funding acquisition. **Kongchang Wei:** Writing – review & editing, Writing – original draft, Visualization, Validation, Supervision, Project administration, Methodology, Investigation, Formal analysis, Conceptualization.

## Funding

This work was supported by Empa's Directorate Board (SKINTEGRITY.CH collaborative research program) and the SNSF Spark grant (CRSK-2_190493).

## Declaration of competing interest

The authors declare that they have no known competing financial interests or personal relationships that could have appeared to influence the work reported in this paper.

## Data Availability

Data will be made available on request.
