# Supplementary material for: Mechanically robust non-swelling cold water fish gelatin hydrogels for 3D bioprinting
Source: Mater Today Bio. 2025 Mar 22;32:101701. doi: 10.1016/j.mtbio.2025.101701 (PMC11986614; doi:10.1016/j.mtbio.2025.101701)
Supplement: Multimedia component 1 [file mmc1.docx]

Supporting information

**Mechanically robust non-swelling cold water fish gelatin hydrogels for 3D Bioprinting**

Tobias Hammer^1^, Ke Yang^2^, Tobias Spirig^1,2^, Barbara Meier-Schiesser^3^, Markus Rottmar^2,*^, Katharina

Maniura-Weber^2^, René M. Rossi^1,*^, Kongchang Wei^1,2, *^

^1^ Empa, Swiss Federal Laboratories for Materials Science and Technology, Laboratory for Biomimetic Membranes and Textiles, Lerchenfeldstrasse 5, St. Gallen, 9014, Switzerland

^2^ Empa, Swiss Federal Laboratories for Materials Science and Technology, Laboratory for Biointerfaces, Lerchenfeldstrasse 5, St. Gallen, 9014, Switzerland

^3^ University Hospital Zurich, Department of Dermatology, Zürich, Zürich, Switzerland

* M. Rottmar, markus.rottmar@empa.ch

* R. Rossi, rene.rossi@empa.ch

* K. Wei, kongchang.wei@empa.ch

**1 Materials**

Materials used in this study include Gelatin from cold water fish skin (Sigma-Aldrich, G7041-500G, Lot. SLCH9658), Phosphate buffered saline (PBS, Sigma-Aldrich, P4417-100TAB), 2-Hydroxy-4′-(2-hydroxyethoxy)-2-methylpropiophenone (Irgacure 2959, Sigma-Aldrich, 410896-10G), Dulbecco's Modified Eagle's Medium (DMEM, Sigma-Aldrich, D5796), Dulbecco's Phosphate Buffered Saline (DPBS, Sigma-Aldrich, D8537-500ML), fetal bovine serum (Sigma-Aldrich, F9665, Lot. 0001655440), penicillin-streptomycin (Sigma-Aldrich, P4458-100ML, Lot. 0000192944), L-glutamine (Sigma-Aldrich, G7513-100ML, Lot. RNBM0086), agarose (VWR, 733-1537), lithium phenyl-2,4,6-trimethyl-benzoylphosphinate (LAP, Sigma-Aldrich, 900889-1G), rhodamine B isothiocyanate (Sigma-Aldrich, R1755-100MG), 10% neutral buffered formalin (Sigma-Aldrich, HT501128), calcein AM (Sigma-Aldrich, 206700-1MG), ethidium homodimer (Sigma-Aldrich, 46043-1MG-F), Cysteine Hydrochloride Monohydrate (Thermo Scientific, Product No. 44889), Ethylenediaminetetraacetic acid disodium salt dihydrate (Sigma-Aldrich, E5134), Bovine Serum Albumin (Sigma-Aldrich, A9647-50G), Cis-5-norbornene-endo-2,3-dicarboxylic anhydride (19.3 g, Sigma-Aldrich, 247634-5G), Collagenase II (Sigma-Aldrich, C2-28-100MG).

**2 Methods**

**2.1 Synthesis of functional gelatin polymers**

Norbornene-functionalized cold water fish gelatin (cfGel-NB):

Cold water fish gelatin (10 g) was dissolved in 100 mL Phosphate buffered saline at 50 °C under constant stirring. Cis-5-norbornene-endo-2,3-dicarboxylic anhydride (19.3 g) was added to the gelatin solution and the pH value of the mixture was adjusted to between 7.5-8.0 by adding NaOH (aq. 1 M) until the solid cis-5-norbornene-endo-2,3-dicarboxylic anhydride was fully dissolved. The reaction was continued for 48 hours with pH value maintained between 7.5-8.0. Subsequently, the reaction was cooled down to room temperature (23 °C) and dialysed against DI water for 1 day and nanopure water for 2 days (Mw. cutoff 6-8kD). A foam-like product (8.5 g) was obtained after freeze-drying and analysed by proton nuclear magnetic resonance (^1^H NMR, **Fig. S1**).

Thiolated cold water fish gelatin (cfGel-SH):

Cold water fish gelatin (20 g) was dissolved in 0.1 M sodium bicarbonate buffer (pH 10, 200 mL) at room temperature under constant stirring. Ethylenediaminetetraacetic acid (EDTA, 87.6 mg) was added to the solution and the mixture was purged with argon gas for 2 minutes. N-acetylhomocystein thiolactone (6.13 g) was added to the solution under argon gas protection. The reaction was continued at room temperature for 12 hours. Afterwards, undissolved solids were removed by filtration. The solution was dialysed against DI water for 1 day and nanopure water for 2 days, concentrated to 150 mL by rotary evaporation, and subsequently freeze-dried, yielding a foam-like product. Analysis was conducted using the Ellman's reagent assay (Thermo Fisher Scientific).

**2.2 Ellman's reagent assay**

To evaluate the degree of functionalization of cfGel-SH, we performed Ellman's reagent assay to quantify the number of sulfhydryl groups within the polymer using cysteine standards. Cysteine standards were prepared by dissolving varying amounts of Cysteine Hydrochloride Monohydrate in Reaction Buffer (0.1 M sodium phosphate, pH 8.0, containing 1 mM Ethylenediaminetetraacetic acid disodium salt dihydrate. The following standard samples were used:

|  | **Volume of Reaction Buffer** | **Amount of Cysteine** | **Final Concentration** |
| --- | --- | --- | --- |
| **Stock Solution** | 2 mL | 5.307 mg | 15 mM |
| **A** | 1.8 mL | 0.2 ml of Stock Solution | 1.5 mM |
| **B** | 0.1 mL | 0.5 mL of A | 1.25 mM |
| **C** | 0.2 mL | 0.4 mL of A | 1.0 mM |
| **D** | 0.3 mL | 0.3 mL of A | 0.75 mM |
| **E** | 0.4 mL | 0.2 mL of A | 0.5 mM |
| **F** | 0.5 mL | 0.1 mL of A | 0.25 mM |
| **G** | 0.1 mL |  | Blank |

Next, a set of test tubes, each containing 10 μL of Ellman's Reagent Solution and 0.5 mL of Reaction Buffer were prepared, followed by the addition of 50 μL of each standard or cfGel-SH solution (0.5 mg mL^-1^, 1 mg mL^-1^, 10 mg mL^-1^, 100 mg mL^-1^ in PBS). After mixing, samples were incubated at room temperature for 15 min before being added in triplicates (100 μL each) into the wells of a 96-well plate (Product No. 92096) and absorbance at 405 nm was measured using a Mithras^2^ LB 943 Multimode Microplate Reader. Values obtained for cysteine standards were plotted to create a standard curve, from which the experimental sample concentrations were determined (**Fig. S2** and **Table S1**).

**2.3 Hydrogel molding with different formulations**

Hydrogel precursor solutions were prepared by dissolving lyophilized cfGel-NB and cfGel-SH in 1×PBS at room temperature through alternating use of vortexing and centrifugation until a clear homogeneous solution was obtained, after which 2-Hydroxy-4′-(2-hydroxyethoxy)-2-methylpropiophenone (Irgacure 2959, stock solution 0.5% (w/v) in UPW) was added to yield the hydrogel precursor solutions with final Irgacure 2959 concentration at 0.05 % (w/v), cfGel-NB and cfGel-SH concentrations as defined for different formulations. For example, for hydrogels containing 5 wt% of polymers at a cfGel-NB to cfGel-SH weight ration of 5:5, the precursor solution would contain 12.5 mg cfGel-NB and 12.5 mg cfGel-SH in 425 μL PBS with 50 μL Irgacure 2959 stock solution.

To form the hydrogels, 40 μL of the solution was cast into a custom-made mold consisting of a rectangular sheet of PDMS of 1 mm thickness containing holes of 6 mm diameter pressed onto a glass slide. To allow for easier removal of crosslinked hydrogels, the glass slide was treated with Sigmacote® (Sigma-Aldrich, SL2). In cases where a flat hydrogel surface was required, the loaded mold was covered with a second PDMS sheet prior UV-irradiation for 2 min at room temperature using a 12W (VL-206.BL, 2×6W, 365 nm) UV light source spaced ~3.5-0 cm away from the mold to ensure the intensity of 7.5-10 mW/cm^2^ throughout the whole mold. Adding 1×PBS between the two PDMS-sheets to lubricate the hydrogels allowed for easier removal of the components of the mold without incurring damage to the gels. We found that using a cell-scraper was the easiest way to remove cfGel-Hydrogels from the glass surface.

The weight ratio between cfGel-NB and cfGel-SH for correct [-NB]:[-SH] stoichiometry is defined as Rs. A formulation with Rs ensures the equal molar concentration between [-NB] and [-SH] groups, thus represents the theoretically optimum formulation for the highest hydrogel modulus under fixed polymer concentration. Since the [-NB] content and [-SH] content of each gram functional polymer is known to be 170 μg and 240 μg, Rs can be calculated as 240/170 (Rs~6:4).

**2.4 Rheological measurements**

Rheological measurements were conducted using an Anton Paar Physica MCR 301 Rheometer equipped with a PP7 parallel plate geometry (diameter 7 mm, Cat No. 10636, without Toolmaster) and a UV light curing system (P-PTD200/GL) in air at 25°C. Oscillatory time-sweep measurements were performed for 4 min at a frequency of 1 Hz and were prepared by adding 60 μL of precursor solution onto the UV light curing stage and lowering the geometry to 1 mm, resulting in the precursor solution filling out the entire space between PP7 plate and the sample stage. Once in place, the hood of the Peltier temperature control system was lowered over the sample and the measurement was started. After 30 seconds of measuring, the precursor solution was exposed to 365nm UV-irradiation (10 mW) using an OmniCure UV curing system (Exfo, Model No. S1000-IB). Irradiation was stopped after 2.5 min and the measurement continued for another 1 min before termination.

Frequency sweep measurements were performed at a frequency of 10-0.01 Hz directly following time-sweep measurements. Strain sweep measurements were conducted after frequency sweep measurements at a frequency of 1 Hz and an increase in strain from 1-1000% until a clear drop in modulus was detected. Steady state viscosity measurements were conducted at 25°C using a CP50 cone plate geometry (0.996 degree, diameter 50 mm).

**2.5 Swelling ratio measurements**

Swelling ratio (SR) was evaluated by determining the change in weight of as-prepared "relaxed" and swollen hydrogels at specific timed intervals. Following separation from the mould, any excess liquid was removed from the hydrogels before their initial weight *W_0_* was recorded. Hydrogels were subsequently placed in 12-well TPP tissue culture test plates (TPP, Product No. 92012) and incubated in either variably concentrated PBS or UPW at 37°C for up to 24 h. The weight of the swollen hydrogels *W_t_* was recorded after 1 h, 2 h, 4 h, 6 h and 24 h by removing them from the respective incubation medium and drying off any excess liquid. The swelling ratio was calculated by applying the following formula:

$$Swelling ratio (SR) \left( \% \right)= \frac{W_{t}-W_{0}}{W_{0}}\times100\%$$

**2.6 Compression measurements**

Disc-shaped hydrogels (6 mm diameter × 1 mm thickness) were exposed to uniaxial compression using an Anton Paar Physica MCR 301 Rheometer in either PBS or UPW at 37°C. Prior to testing, samples were incubated for 1 h in PBS or UPW at 37°C in a laboratory warming cabinet (Labocult, Model H7 70) to prevent excessive swelling during the measurements, which is especially relevant for UPW-exposed hydrogels. Further swelling after 1 h was prevented by removing most of the incubation medium, leaving only enough to maintain a humid environment to counteract dehydration as samples were kept at 37°C until being measured. Samples were incubated and kept in 12-well tissue culture test plates (TPP, Product No. 92012). For compressive measurements, samples were first transferred into a flat-bottomed plastic petri dish and the diameter was recorded using an Aerospace 150 mm Digital Vernier Caliper. Next, the petri dish was placed on the lower plate of the rheometer while the upper plate slowly (10 μm/s) approached the sample until contact was established, indicated by an increase in the detected normal force. The initial measurement position was then set to be slightly (one or two measurement points) above the first point of positive force detection to avoid excessive preliminary compression. Following contact establishment, the petri dish was filled with either PBS or UPW until the PP25 parallel plate (diameter 25 mm, Cat No. 79044) was completely submerged. Both incubation media as well as the PP25 plate were heated to 37°C to prevent temperature fluctuations during the measurements. Samples were allowed to equilibrate for 5 min in the incubation medium before compressive tests were started. Compression and relaxation were performed at a constant speed of 50 μm/s and frequency of 0.001 Hz for various amounts of time corresponding to 80% compressive strain. The amount of time *t* required was determined using the following formula:

$$t=\frac{\left( h_{i}-h_{d} \right)\times0.8}{0.05},$$

where *h_i_* is the initial height used as the measurement position and *h_d_* the height of the petri dish to be subtracted to obtain the original height of the hydrogel sample *h_s_*. Multiplying the original height of the sample by 0.8 reveals the required distance the geometry has to descend in order to achieve 80% compression. By further dividing the distance by the travel speed of 0.05 mm/s, we obtain the amount of time needed per interval of compression or unloading. For measurements, four consecutive cycles of compression were executed without intermediate waiting times between cycles. Hysteresis loss was calculated by integrating the area between loading and unloading curves using Origin.

To determine the Young's modulus of PBS- and UPW-swollen cfGel-Hydrogels, stress *σ* and strain *ε* were calculated using the following formulas:

$$\sigma=\frac{F_{t}\times(h_{t}-h_{d})}{{\pi r}^{2}\times h_{s}},$$

$$\varepsilon=\frac{h_{i}-h_{t}}{h_{s}}\times100\%,$$

where *F_t_* and *h_t_* represent the measured force and height (gap size) at any given time during the measurement and *r* the original radius of the sample.

For residual strain compression, measurement procedures were slightly altered. Instead of defining the interval time for loading and unloading needed based on the travel speed of the PP25 plate, we defined the upper and lower limits of gap size corresponding to the respective percentage of compression, with loading and unloading sequences being separated into different measurement intervals. As soon as the lower limit of the gap size, which corresponded to 80% compression, was reached, the unloading interval would start and lift the geometry to the upper gap size limit, which corresponded to the respective residual strain.

**2.7 Tensile mechanical measurements**

Tensile testing was performed using a BioTester 3000 Biaxial Test System (CellScale) equipped with a 0.5 N load cell and a heatable fluid chamber. Rectangular samples with 20 mm length × 6.5 mm width × 1 mm thickness were secured with two mechanical clamps set apart at a distance of ~6.5 mm and lowered into the pre-heated fluid bath (DPBS). Samples were incubated in PBS at 37°C for at least 1 h prior to mounting and afterwards kept at room temperature until used. To prevent damage to the samples during mounting, hydrogels were placed on a supportive structure consisting of a piece of paper until submerged. Afterwards, parts of the supportive structure were removed so as to not interfere with the measurement and samples were given another ~5 min of equilibration time in the heated fluid bath (37 °C) before stretching. Samples were stretched for 5 cycles at a speed of 1 mm/s at increasing intervals of 1 mm (i.e. 5 cycles of 1 mm stretch – 5 cycles of 2 mm stretch – 5 cycles of 3 mm stretch…) until failure. For the first cycle of each interval, a pre-load of 1.5 mN was applied before stretching. After each interval, the distance was reset to the original position prior to pre-loading.

**2.8 Cell culture**

Primary human dermal fibroblasts (HDFs, CELLnTEC, Lot. EB1104281, pooled juvenile donors) were cultured in Dulbecco's Modified Eagle's Medium (DMEM, Sigma-Aldrich, D5796) supplemented with 10% (v/v) fetal bovine serum (Sigma-Aldrich, F9665, Lot. 0001655440), 1% penicillin-streptomycin (Sigma-Aldrich, P4458-100ML, Lot. 0000192944), and 1% L-glutamine (Sigma-Aldrich, G7513-100ML, Lot. RNBM0086) in a humidified environment at 5% CO_2_ and 37°C. HDFs were used up to passage 10. Cell encapsulations for cytocompatibility evaluation were done with passages 4, 6, and 7. Bioprinting experiments were conducted with passages 4, 5, 6, and 9. Cells were cultured in standard tissue culture plastic flasks (TPP, Product No. 90076) with media changes being conducted twice a week until 80%-100% confluency. Following trypsinization, cells were collected in cell culture medium, pelleted and reconstituted in cfGel-Hydrogel precursor solution at a density of 2'000'000 cells/mL for both cell encapsulation and bioink preparation purposes. Cell-laden hydrogels were molded in the same way as described in section "2.3 Hydrogel molding with different formulations", with the abovementioned amount of cells suspended in the precursor solutions.

**2.9 Three dimensional (3D) bioprinting**

Agarose suspension baths were prepared in accordance to previously published descriptions [1]. Specifically, agarose (VWR, 733-1537) was added to UPW at a concentration of 0.5% (w/w) and autoclaved at 120°C. Directly following autoclaving, the still hot agarose solution was placed on a stir plate and sheared at 700 rpm until it cooled to room temperature and was subsequently stored at 4°C until further use. To prepare suspension baths, the 0.5% (w/w) agarose solution was diluted with sterile Dulbecco's Phosphate Buffered Saline (DPBS, Sigma-Aldrich, D8537-500ML) to a concentration of 0.25% (w/w) and centrifuged at 500 rpm for 5 min before being added to 12-well plates.

Both cell-laden and cell-free constructs were printed using a Bio X6 extrusion printer (CELLINK). Inks used for cell-free printing consisted of 2.5% (w/w) cfGel-NB and 2.5% (w/w) cfGel-SH with 10% (w/w) lithium phenyl-2,4,6-trimethyl-benzoylphosphinate (LAP, 0.5% (w/w), Sigma-Aldrich, 900889-1G) as photoinitiator, 10% (w/w) rhodamine B isothiocyanate stock solution (1% (w/w), Sigma-Aldrich, R1755-100MG) for visualization, and DPBS as the solvent. Cell-free constructs with 9 mm length × 9 mm width × 0.6 mm thickness were printed in a rectilinear pattern with 8% infill at a speed of 10 mm/s and an extrusion rate of 1.5 μL/s using a 27 gauge needle. For each independent repeat, four constructs were successively printed and crosslinked at the same time using an external UV-source (VILBER, VL-206.BL, 2×6W, 365 nm). After printing, constructs were imaged using a confocal microscopy system (Zeiss LSM780) while still encased in the support bath to record the as-printed dimensions. Afterwards, the support bath was removed and the samples were washed 3× with and subsequently submerged in DPBS before another round of image acquisition. Samples were then incubated at 37°C for two weeks in a laboratory warming cabinet (Labocult, Model H7 70) without CO_2_ control and imaged at day 7 and 14.

For bioprinting, bioinks were prepared by dissolving 2.5% (w/w) cfGel-NB and 2.5% (w/w) cfGel-SH together with 10% (w/w) LAP (0.5% (w/w)) and HDFs (2'000'000 cells/ml) in DPBS. Cell-laden constructs with 6 mm length × 6 mm width × 1 mm thickness were printed in a grid pattern with 24% infill at a speed of 10 mm/s and an extrusion rate of 1.5 μL/s using a 27 gauge needle. For each independent repeat, four constructs were successively printed and crosslinked at the same time using an external UV-source (VILBER, VL-206.BL, 2×6W, 365 nm). Immediately after printing, support baths were removed and samples were washed 3× with sterile DPBS before being submerged in DMEM cell culture medium supplemented with 10% (v/v) FBS, 1% (v/v) penicillin/streptomycin and 1% (v/v) L-glutamine and incubated at 37°C and 5% CO_2_. Images were taken at day 1 and 7 for live/dead staining, and day 7 and 14 for immunostainings respectively.

**2.10 Immunofluorescence staining and analysis**

Immunofluorescence analysis was conducted by fixating cfGel-Hydrogels containing encapsulated HDFs in 10% neutral buffered formalin (Sigma-Aldrich, HT501128) for ~30 min followed by permeabilization with 0.1% Triton X-100 (Sigma-Aldrich, T8787-100ML) for ~15 min. Samples were subsequently washed with DPBS 3× for 1 min before being either stored at 4°C or directly processed further. Blocking was performed in 3% bovine serum albumin (BSA in DPBS, Sigma-Aldrich, A9647-50G) for at least 1h (ranging up to ~5h) at room temperature or o/n at 4°C before samples were incubated in primary antibody solution o/n at 4°C. After secondary antibody incubation for 1-1.5h at room temperature in the dark, samples were washed 3× with DPBS for 1 min. All antibodies were diluted by a factor of 1:200 in DPBS containing 1% fetal bovine serum, with DAPI being diluted by 1:1000. For live/dead staining, samples were incubated with cell culture medium containing 0.1% (v/v) calcein AM (stock: 1mM in DMSO, Sigma-Aldrich, 206700-1MG) and 0.1% (v/v) ethidium homodimer (stock: 1mM in DMSO, Sigma-Aldrich, 46043-1MG-F) for ~1h at 37°C followed by washing in DPBS 3× for 1 min. Samples were imaged in DPBS.

**2.11 Collagenase II degradation assay**

To evaluate the degree of enzymatic degradation for both cfGel-Hydrogels as well as GelMA-based hydrogels, disc-shaped hydrogels (5 wt%) of 6 mm diameter and 1 mm thickness were incubated in 12-well TCP plates with 3 mL of Collagenase II (Sigma-Aldrich, C2-28-100MG, 125 to 250 Mandl units per milligram of powdered substance) at a concentration of 0.01 mg/ml (~2 U/ml activity) in PBS at 37°C. Prior to the start of the degradation assay, hydrogels were incubated in PBS at 37°C overnight to prevent the influence of swelling/de-swelling-mediated changes to hydrogel weight during the assay. Hydrogels were removed from the Collagenase II solution at specific time points, blotted dry and weighted. The residual mass of the hydrogels was calculated using the following equation:

$Mass \left( \% \right) = \frac{W_{t}}{W_{0}}\times100$

**2.12 Printing resolution evaluation**

The printing resolution of both cell-inclusive and cell-free prints was evaluated using the Pr score metric [2]. The Pr score was calculated using the formula:

$$\Pr= \frac{L^{2}}{16A}$$

where L represents the perimeter and A the area of a pore. In case of an ideal, rectangular pore, the Pr score equates to 1. Inks with under- and over-gelation properties usually display Pr scores <1 and >1 respectively. As an additional measure of precision, we calculated the area uniformity of the pores using the following formula:

$$Area Uniformity = 1- \frac{\sigma_{Pore area}}{\mu_{Pore area}}$$

where $\sigma_{Pore area}$ represents the standard deviation of the pore areas and $\mu_{Pore area}$ represents the mean of the pore areas respectively.

**3. Supplementary data**


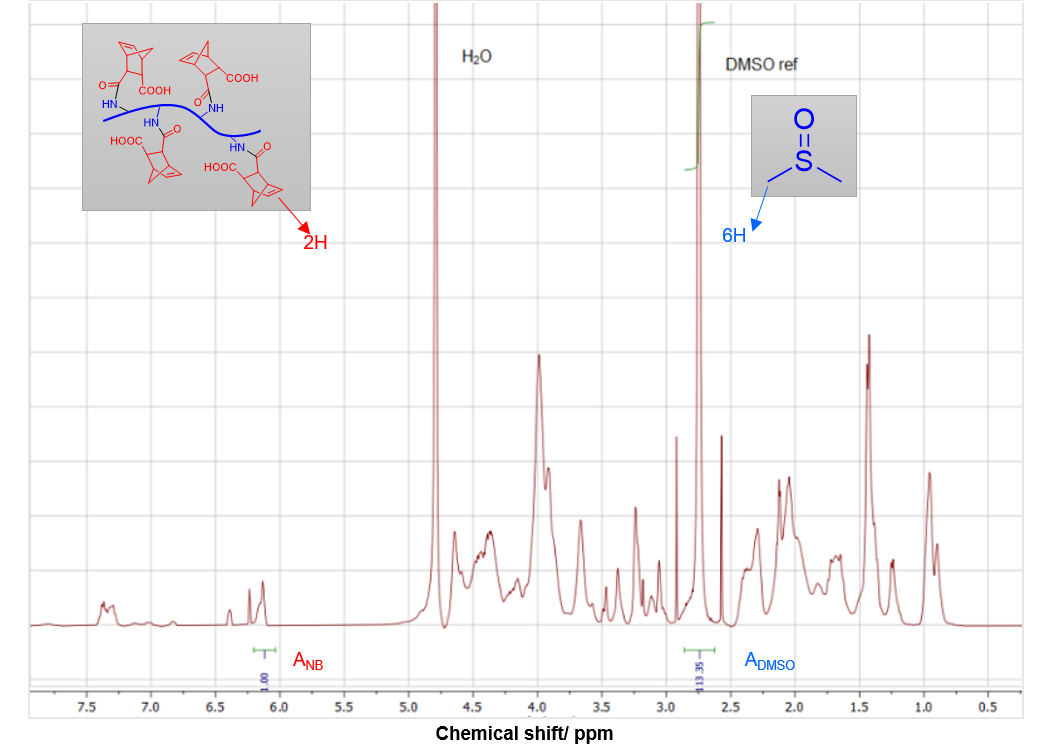


**Figure S1.** ^1^H NMR of cfGel-NB functional polymer. The content of norbornene [-NB] groups was calculated by comparing the integration of the proton signals between unsaturated double bonds (A_NB_) and a DMSO external reference (A_DMSO_). With M_1_ mg cfGel-NB and M_2_ mg DMSO dissolved in the sample (DMSO-d6 solution), the NB content (C_NB_, mol) can be calculated using the following formula:

$$\frac{A\text{NB}}{\text{A}\text{DMSO}} = \frac{(2 x \text{C}\text{NB})}{6 \times\frac{\text{M}\text{2}}{\text{M}\text{DMSO}}}$$

where, according to the spectrum,

$$\frac{\text{A}\text{NB}}{\text{A}\text{DMSO}} = \frac{1}{113.35}$$

the molecular weight of DMSO is M_DMSO_ = 78.13 g/mol, and M_1_ and M_2_ are known from the experiment (50mg and 5 mg respectively). Therefore, the content of norbornene per gram polymer is:

$$\frac{\text{C}\text{NB}}{\text{M}\text{1}} = 170 \text{μmol}$$

**
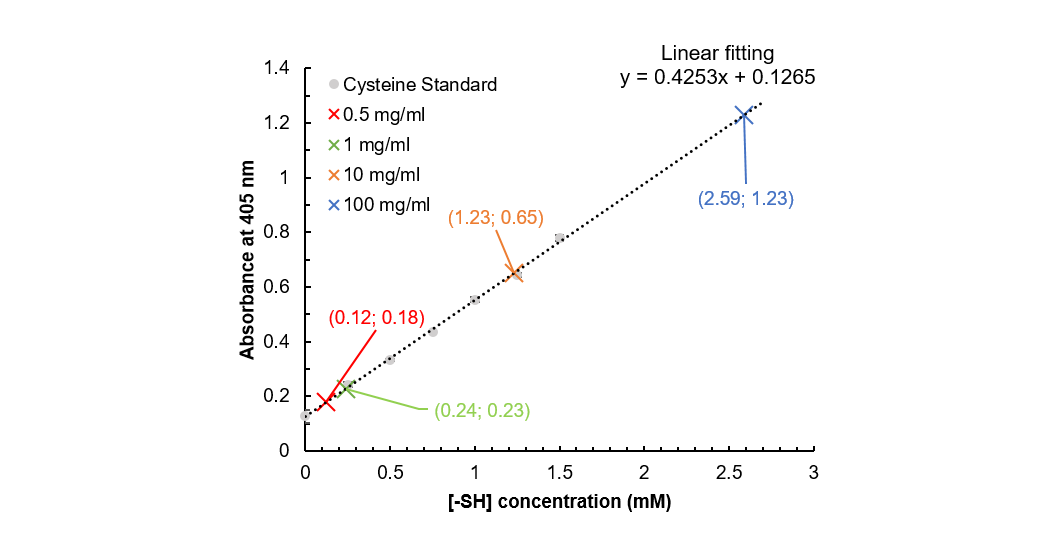
**

**Figure S2.** Thiol contents of cfGel-SH were determined via Ellman's reagent assay using a cysteine standard (Methods 2.2). Using lower concentrations of cfGel-SH (e.g. 0.5 mg/ml and 1 mg/ml) yielded similar values regarding the [-SH] content within the polymer, while higher concentrations resulted in significantly reduced values (Table S1). A likely cause could be the formation of disulphide bonds and the reduction of free sulfhydryl groups available to interact with the Ellman's reagent in concentrated solutions of cfGel-SH.

**Table S1.** Measurement of [–SH] contents at four different cfGel-SH concentrations.

| cfGel-SH  (mg/mL) | [-SH]  (mM) | [-SH] content  (μmol/g polymer) |
| --- | --- | --- |
| 0.5 | 0.12 | 245.317 |
| 1 | 0.24 | 241.006 |
| 10 | 1.23 | 123.246 |
| 100 | 2.59 | 25.876 |


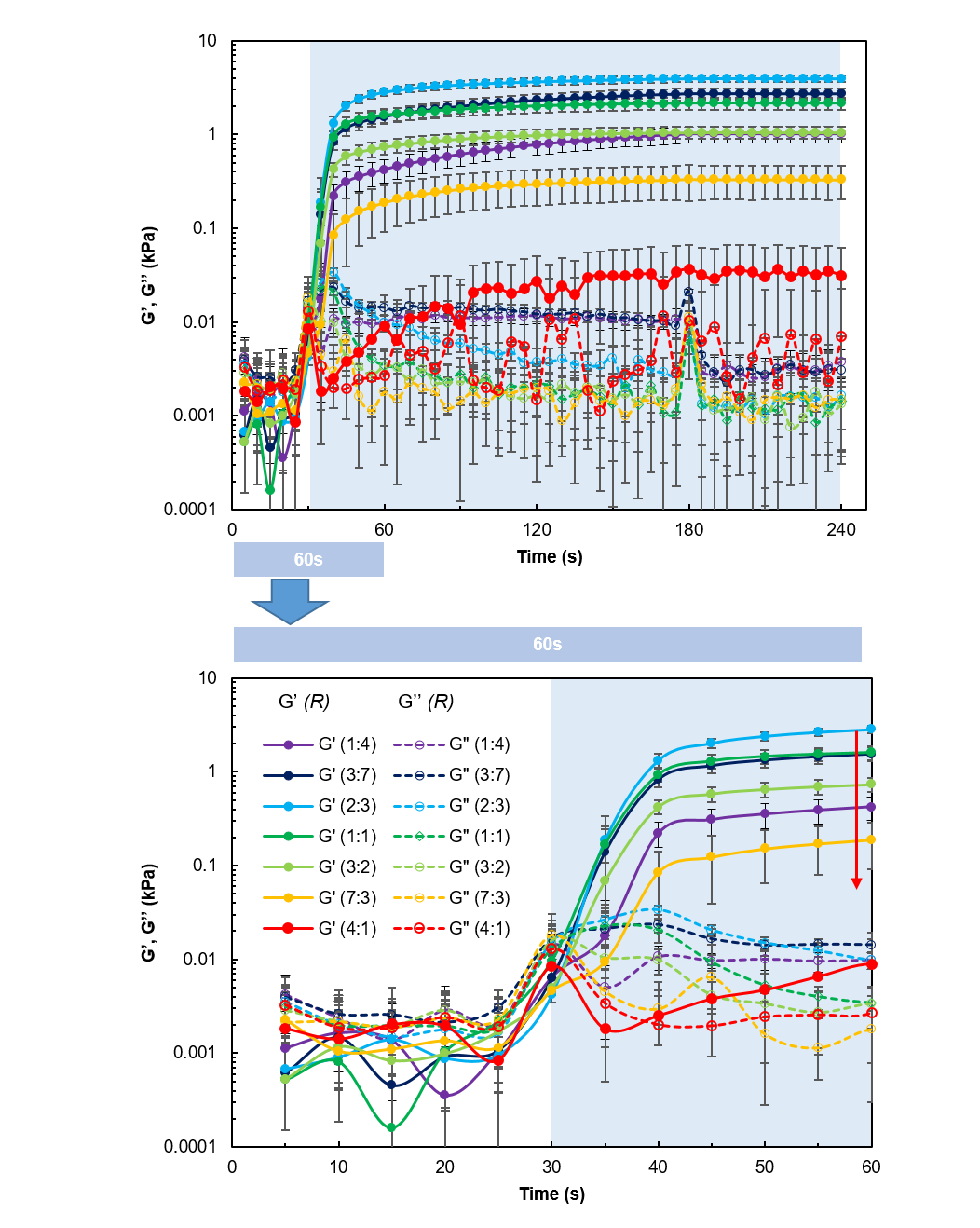


**Figure S3.** Rapid gelation of 5 wt% cfGel-Hydrogels with different ratios (R, by weight) between cfGel-NB and cfGel-SH. Blue area denotes the timespan of UV-exposure.


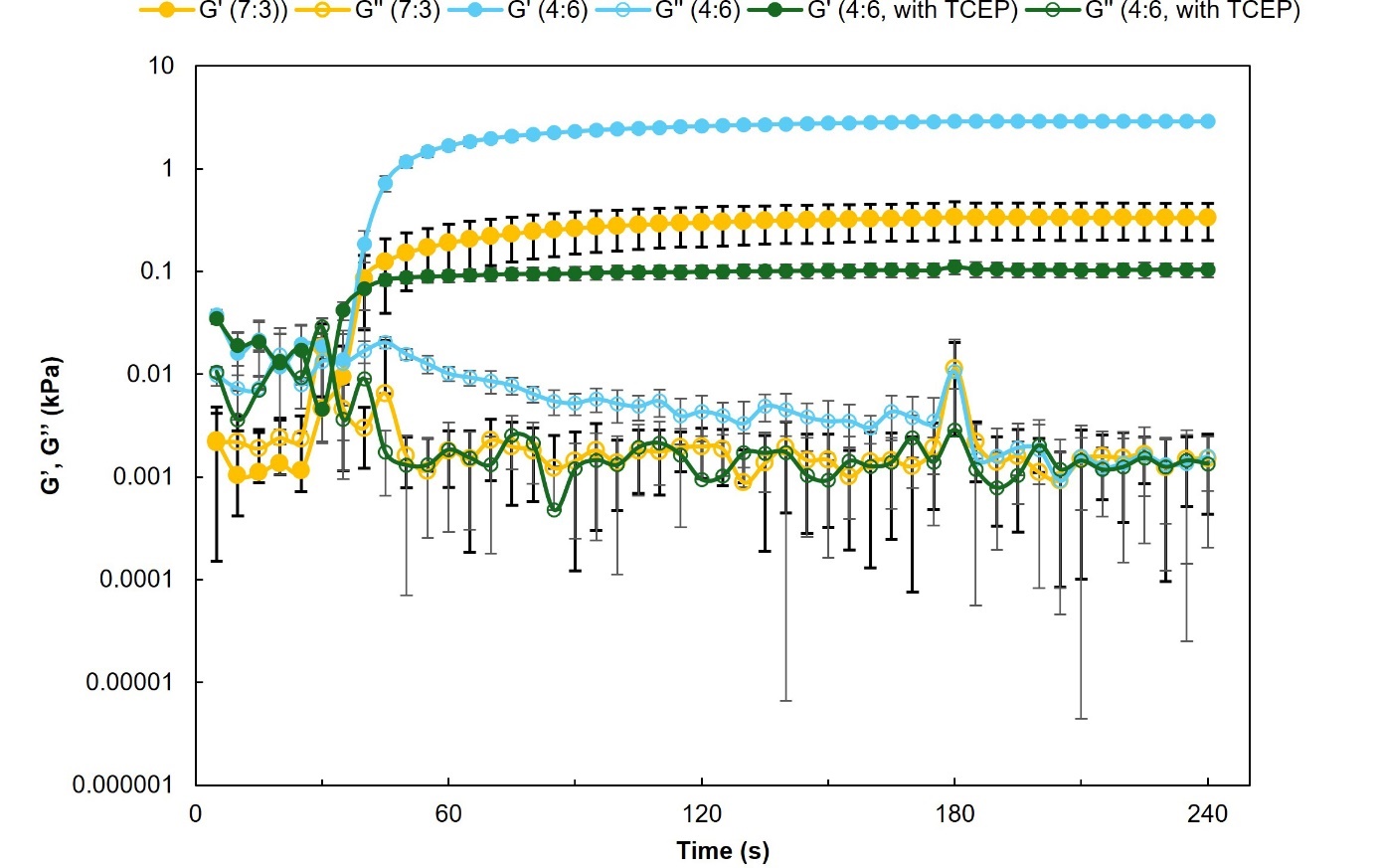


**Figure S4.** A comparison between cfGel-Hydorgel (R = 4:6) curing profiles with or without TCEP as the reducing agent for preventing disulfide crosslinking (*n = 3*). (for the samples with TCEP: ~1.8 mg TCEP was dissolved in 0.25 mL precursor solution, to give the molar ratio between thiol and TCEP as ~1:2)

**Note:**

With a fixed total concentration of cfGel polymers at 5 wt%, the theoretical thiol-ene click crosslinking points for the formulation R = 4:6 (in presence of TCEP) depends on the amount of norbornene groups (C_N(4:6)_). Given a precursor solution volume (V mL), C_N(4:6)_ can be estimated as:

C_N(4:6)_= (5% × V × 0.4 × 170) μmol = 3.4 × V μmol

This is similar to that of the formulation R = 7:3 (without TCEP), where norbornene groups are excessive and the theoretical thiol-ene click crosslinking points could be estimated by the amount of thiol groups:

C_N(7:3)_= (5% × V × 0.3 × 240) μmol = 3.6 × V μmol

Therefore, if disulfide crosslinking were prohibited by TCEP, the G' of the thiol-dominant cfGel-Hydrogels (R = 4:6, with TCEP) was expected to be close to the norbornene-dominant cfGel-Hydrogels (R = 7:3, without TCEP). The rheological measurement (Fig. S4) was in good agreement with this calculation, thus confirming the existence of disulfide crosslinks in cfGel-Hydrogels (R = 4:6, without TCEP) and their contribution to the mechanical properties of the respective hydrogels.


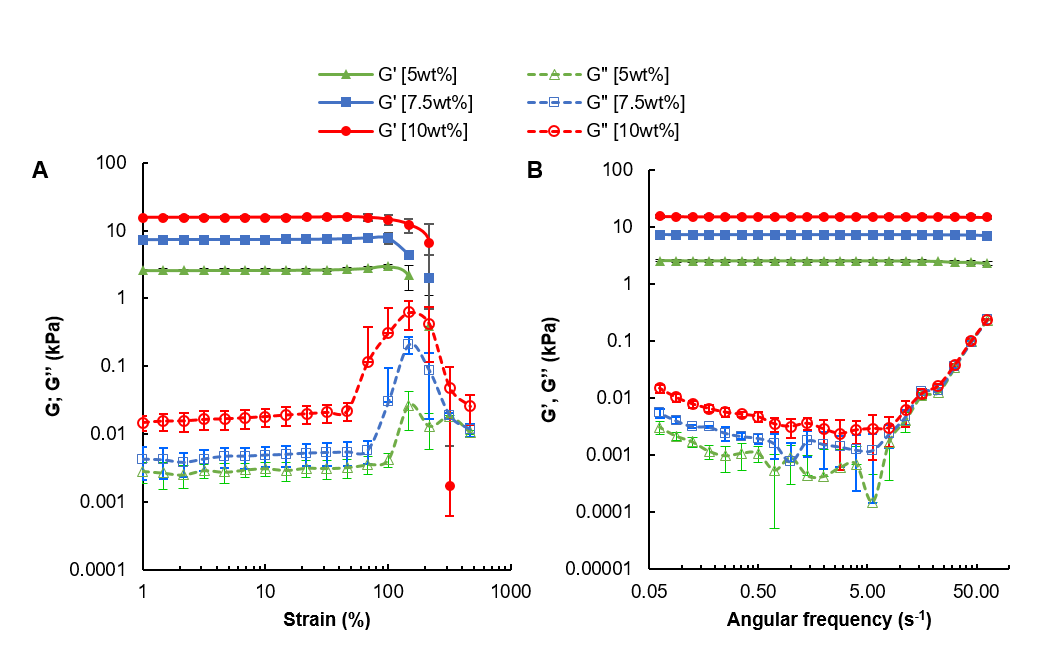


**Fig. S5** Oscillatory strain (**A**) and frequency (**B**) sweep measurements of cfGel-Hydrogels (R = 5:5) at different total cfGel concentrations (*n = 3*).

**
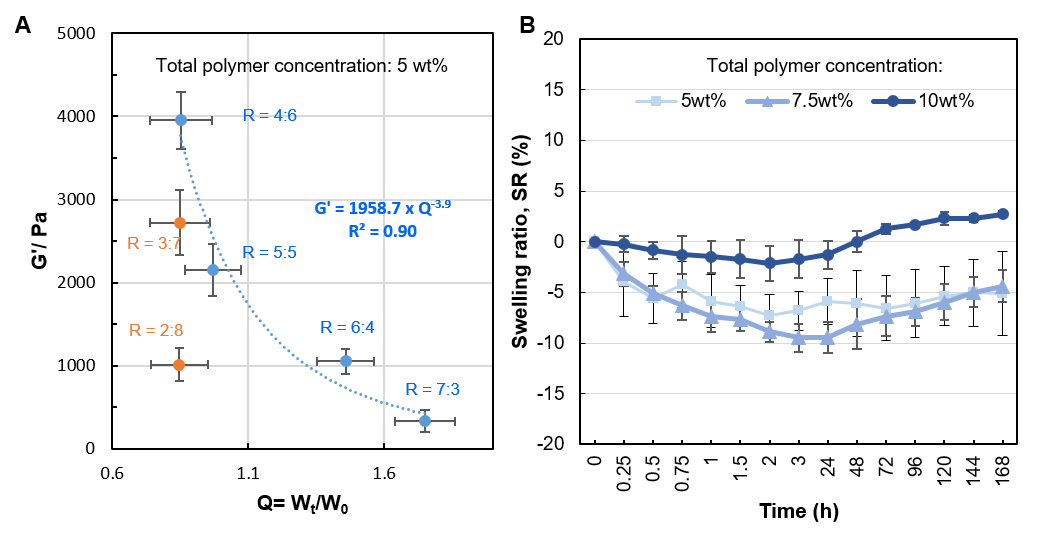
**

**Fig. S6 (A)** Correlation between volume swelling ratio (Q) and storage modulus (G') of cfGel-Hydrogels with 5 wt% fixed total polymer content and varying weight ratio between cfGel-NB and cfGel-SH (R). **(B)** Detailed timeline of swelling ratio of cfGel-Hydrogels with varying polymer concentration (fixed R = 5:5) as measured by changes in weight. Total polymer concentration of cfGel-Hydrogel precursor formulations shows no significant effect on swelling properties of resultant gels. (*n=3*)

Note: Due to the low polymer content of cfGel-Hydrogels (5 wt%), volume swelling ratio (Q) was estimated by hydrogel weight at swollen state (W_t_) and as-prepared state (W_0_), Q= W_t_/W_0_. According to the mass swelling ration SR = (W_t_-W_0_)/W_t_, Q was calculated as Q = 1/(1-SR).

The correlation of storage modulus G' of the in situ formed hydrogels with Q appeared to follow a scale law G' $\propto$ Q^-3.9^ within the range of R from 7:3 to 4:6 (blue data points, Fig. S5A). However, with excessive cfGel-SH at R = 3:7 and 2:8, (orange data points, Fig. S5-A), de-swelling of the cfGel-Hydrogels was not dictated by the initial G' of the in situ formed hydrogels. This indicates that, the de-swelling could be induced by slow post-formation reactions, e.g. disulphide bond formation.

**
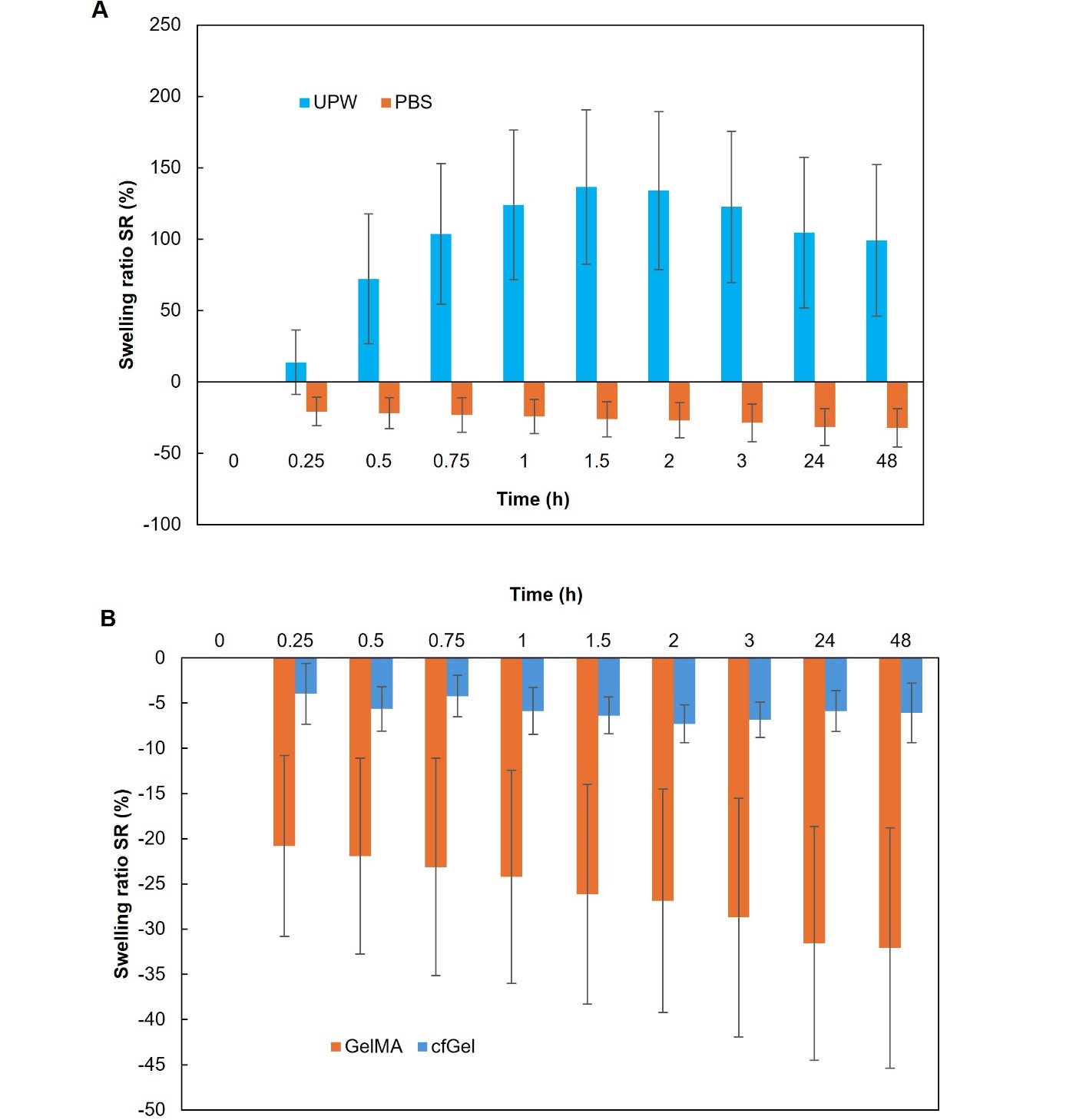
**

**Fig. S7 (A)** Swelling ratio of GelMA-based hydrogels (5 wt%) in PBS and UPW at 37°C recorded over a period of 48 h (*n = 3*). (**B**) Comparison between swelling ratios of 5 wt% cfGel-Hydrogels and GelMA-based hydrogels in PBS at 37°C over a period of 48 h.

**
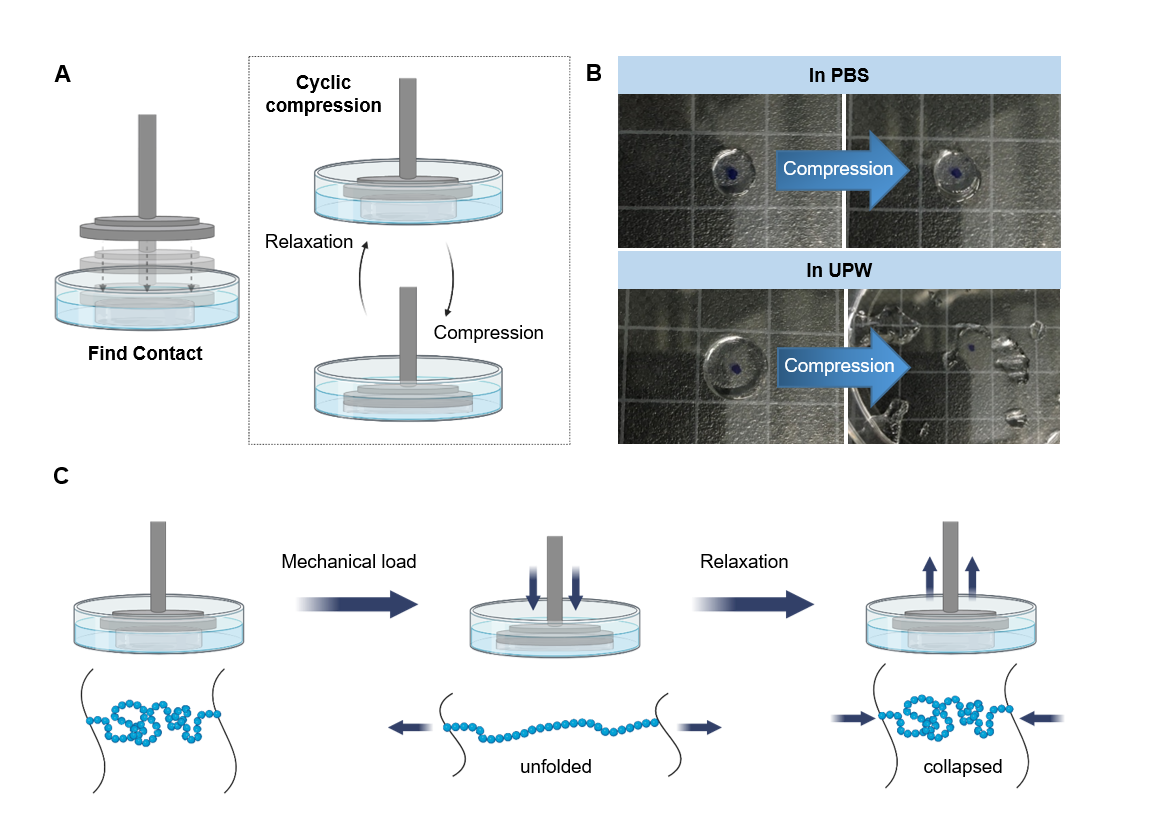
**

**Fig. S8** Cyclic compression test of cfGel-Hydrogel in PBS or ultrapure water (UPW). (**A**) Schematic illustration of the measurement procedure. (**B**) Digital photos of cfGel-Hydrogels before and after cyclic compressions with 80% peak strain. (**C**) Schematic illustration of polymeric conformational change during compressive loading and unloading procedures.


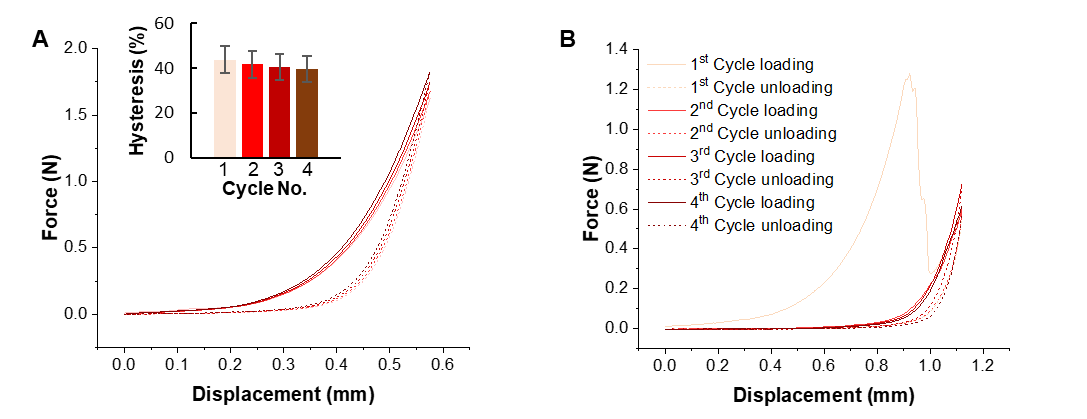


**Figure S9**. Mechanical compression of GelMA-based hydrogels (5 wt%) in PBS (**A**) or UPW (**B**) (*n = 3*).


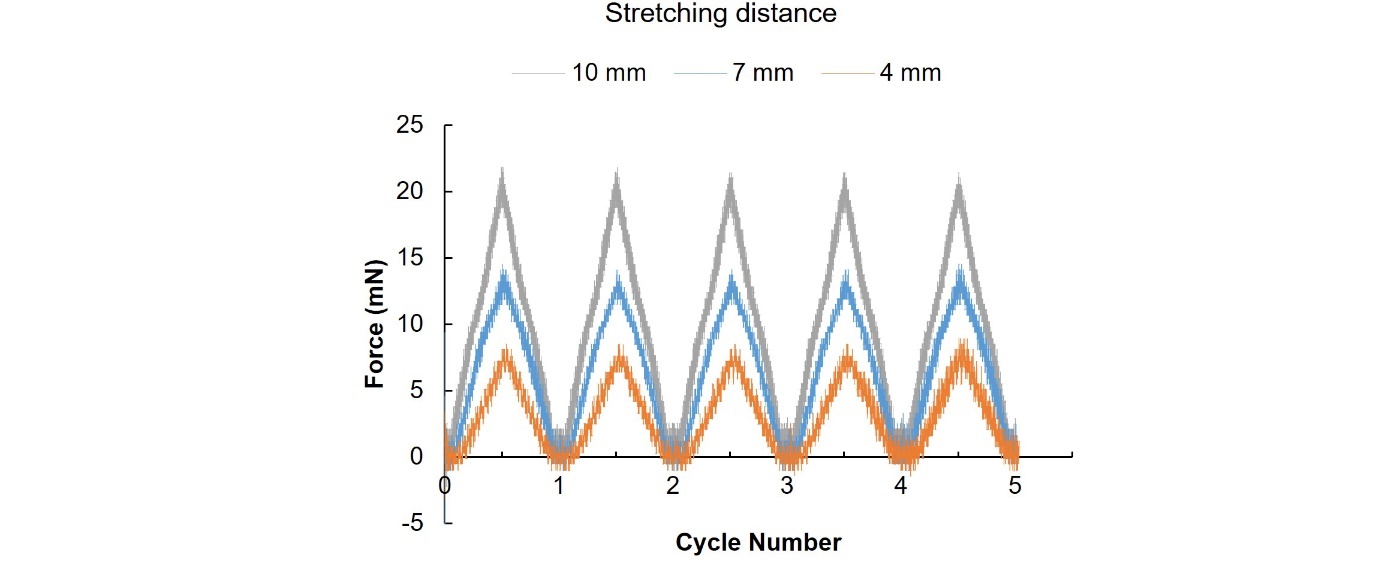


**Fig. S10** Cyclic tensile testing of cfGel-Hydrogels. Samples (5 wt% total polymer concentration, R = 5:5, l = 20 mm, w = 6.5 mm, h = 1 mm) were secured with two mechanical clamps set apart at a distance of ~6.5 mm and stretched at increasingly larger intervals until failure.


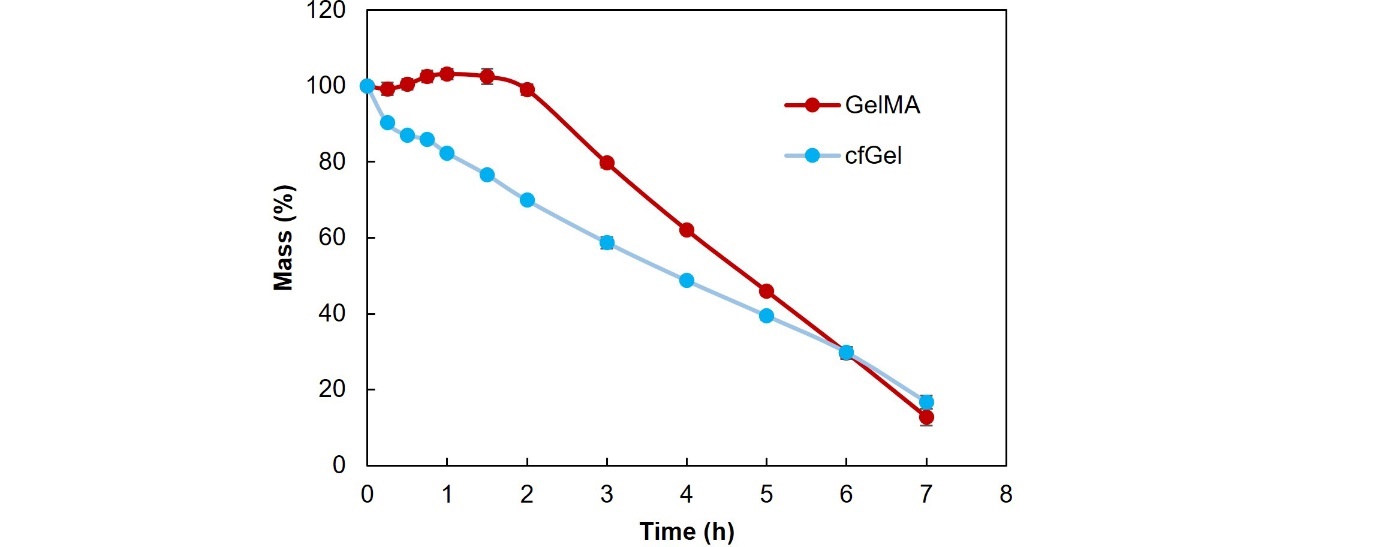


**Fig. S11** Enzymatic degradation of cfGel-Hydrogels and GelMA-based hydrogels by Collagenase II (~2 U/ml). Both types of hydrogels lost more than 80 % of their original weight after 7 h of incubation (*n = 3*).


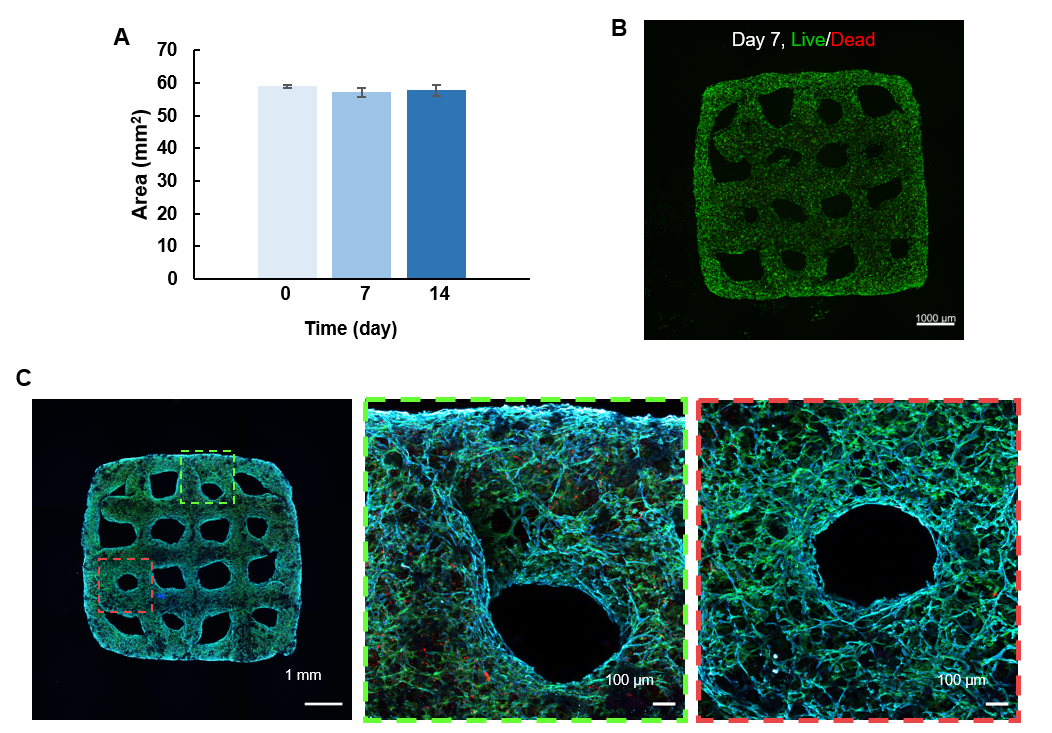


**Fig. S12 (A)** Measured area of imaged grids at different time points (*n = 3*, agarose-suspended samples could not be measured properly and were therefore left out). (**B**) Live/Dead staining image of cell-laden printed constructs on day 7. (**C**) Left: the "Top view" image in Fig. 7B-iii (day 14 immunofluorescence image); middle and right: Zoom-in images of the detailed printed features from left panel.

**
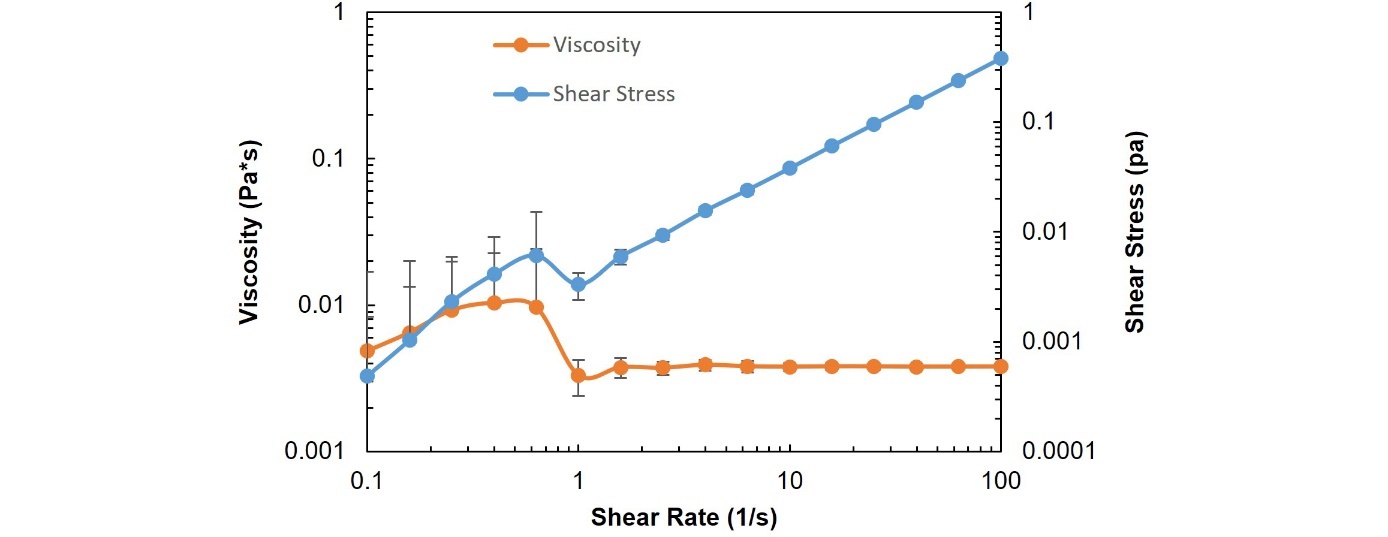
**

**Fig. S13** Steady state viscosity measurements of 5 wt% cfGel precusor solutions (*n = 3*). Precursor solutions of this concentration were used in the printing of cell-free and cell-inclusive constructs. It should be noted that due to the low viscosity, measurements below the shear rate of 1 (1/s) were not reliable. The viscosity (~0.003 Pa·s) of this solution was determined by measurements above this shear rate.


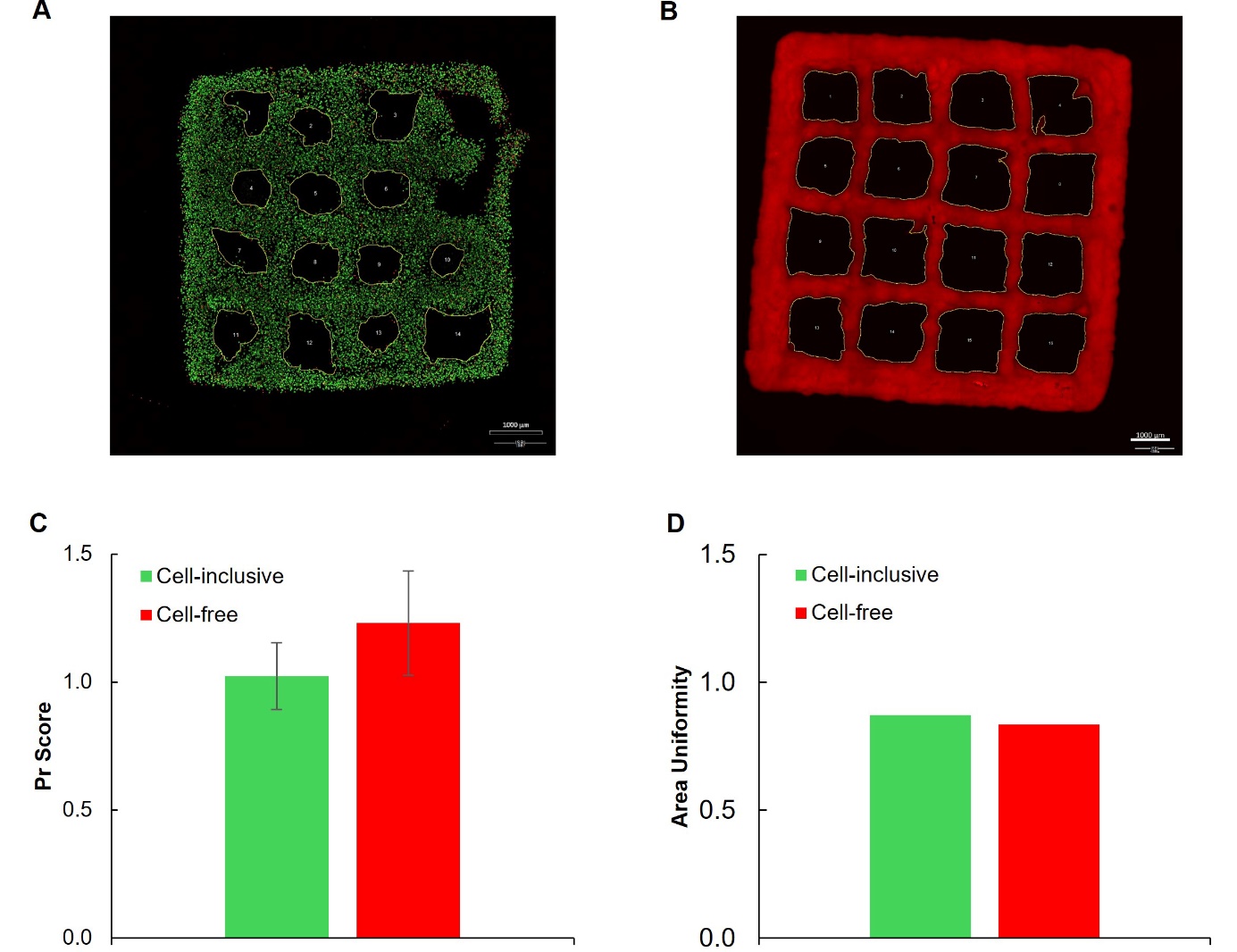


**Fig. S14** Semi-quantitative analysis of 3D bioprinting with 27G needle (see Methods section). For both cell-inclusive (**A**) and cell-free (**B**) constructs, Pr scores are close to 1, despite showing non-rectangular morphology at times (**C**). This might result from the pores of cell-inclusive constructs being both more circular in shape as well as demonstrating a non-smooth perimeter. Cell-free constructs demonstrate Pr scores greater than 1, which would usually indicate over-gelation of the ink, though in this case, it could be caused by the rough surface texture that was induced by diffusion of the ink into the support bath. Pore area uniformity was found to be <1 for both types of constructs (**D**).

**4 Reference**

[1] M. E. Prendergast, and J. A. Burdick, Computational Modeling and Experimental Characterization of Extrusion Printing into Suspension Baths. *Adv. Healthc. Mater.* 11 (7) (2022) e2101679. 10.1002/adhm.202101679

[2] Navara, Adam M., et al. "A dual-gelling poly (N-isopropylacrylamide)-based ink and thermoreversible poloxamer support bath for high-resolution bioprinting." *Bioactive Materials* 14 (2022): 302-312.
